# Epigenetic clock for skin and blood cells applied to Hutchinson Gilford Progeria Syndrome and *ex vivo* studies

**DOI:** 10.18632/aging.101508

**Published:** 2018-07-26

**Authors:** Steve Horvath, Junko Oshima, George M. Martin, Ake T. Lu, Austin Quach, Howard Cohen, Sarah Felton, Mieko Matsuyama, Donna Lowe, Sylwia Kabacik, James G. Wilson, Alex P. Reiner, Anna Maierhofer, Julia Flunkert, Abraham Aviv, Lifang Hou, Andrea A. Baccarelli, Yun Li, James D. Stewart, Eric A. Whitsel, Luigi Ferrucci, Shigemi Matsuyama, Kenneth Raj

**Affiliations:** 1 Department of Human Genetics, David Geffen School of Medicine, University of California Los Angeles, Los Angeles, CA 90095, USA; 2 Department of Biostatistics, Fielding School of Public Health, University of California Los Angeles, Los Angeles, CA 90095, USA; 3 Department of Pathology, University of Washington, Seattle, WA 98195, USA; 4 Department of Clinical Cell Biology and Medicine, Graduate School of Medicine, Chiba University, Chiba, Japan; 5 Elizabeth House, Warlingham, Surrey CR6 9LF, United Kingdom; 6 Department of Dermatology, Oxford University Hospitals NHS Foundation Trust, Oxford OX3 7LJ, United Kingdom; 7 Department of Medicine, Case Western Reserve University, Cleveland, OH 44106, USA; 8 Radiation Effects Department, Centre for Radiation, Chemical and Environmental Hazards, Public Health England, Chilton, Didcot, Oxfordshire, OX11 0RQ, United Kingdom; 9 Department of Physiology and Biophysics, University of Mississippi Medical Center, Jackson, MS 39216, USA; 10 Public Health Sciences Division, Fred Hutchinson Cancer Research Center, Seattle, WA 98109, USA; 11 Institute of Human Genetics, Julius Maximilians University, Würzburg, Germany; 12 Center of Development and Aging, New Jersey Medical School, Rutgers State University of New Jersey, Newark, NJ 07103, USA; 13 Center for Population Epigenetics, Robert H. Lurie Comprehensive Cancer Center and Department of Preventive Medicine, Northwestern University Feinberg School of Medicine, Chicago, IL 60611, USA; 14 Laboratory of Environmental Epigenetics, Departments of Environmental Health Sciences and Epidemiology, Columbia University Mailman School of Public Health, New York, NY 10032, USA; 15 Departments of Genetics, Biostatistics, Computer Science, University of North Carolina, Chapel Hill, NC 27599, USA; 16 Department of Epidemiology, Gillings School of Global Public Health, University of North Carolina, Chapel Hill, NC 27599, USA; 17 Department of Medicine, School of Medicine, University of North Carolina, Chapel Hill, NC 27516, USA; 18 Longitudinal Studies Section, Translational Gerontology Branch, National Institute on Aging, National Institutes of Health, Baltimore, MD 21224, USA; 19 Department of Pathology and Pharmacology, Case Comprehensive Centre, Case Western Reserve University, Cleveland, OH 44106, USA

**Keywords:** epigenetics, fibroblasts, DNA methylation, progeria, Hutchinson-Gilford

## Abstract

DNA methylation (DNAm)-based biomarkers of aging have been developed for many tissues and organs. However, these biomarkers have sub-optimal accuracy in fibroblasts and other cell types used in *ex vivo* studies. To address this challenge, we developed a novel and highly robust DNAm age estimator (based on 391 CpGs) for human fibroblasts, keratinocytes, buccal cells, endothelial cells, lymphoblastoid cells, skin, blood, and saliva samples. High age correlations can also be observed in sorted neurons, glia, brain, liver, and even bone samples. Gestational age correlates with DNAm age in cord blood. When used on fibroblasts from Hutchinson Gilford Progeria Syndrome patients, this age estimator (referred to as the skin & blood clock) uncovered an epigenetic age acceleration with a magnitude that is below the sensitivity levels of other DNAm-based biomarkers. Furthermore, this highly sensitive age estimator accurately tracked the dynamic aging of cells cultured *ex vivo* and revealed that their proliferation is accompanied by a steady increase in epigenetic age. The skin & blood clock predicts lifespan and it relates to many age-related conditions. Overall, this biomarker is expected to become useful for forensic applications (e.g. blood or buccal swabs) and for a quantitative *ex vivo* human cell aging assay.

## Introduction

While our arsenal of potential anti-aging interventions is brimming with highly promising candidates that delay aging in model organisms, it remains to be seen whether these interventions delay aging in humans. The relatively slow pace of this stage is primarily due to the fact that while efficacy of age-related interventions can be reasonably tested in short-lived model organisms, they are not quickly testable in humans, who live much longer. Apart from the impractical measure of human life-span, it is not immediately obvious how the efficacy of an intervention on human aging can be ascertained. To address this challenge, robust biomarkers of aging that are equally effective in *in vivo* as well as *ex vivo* studies are required. These biomarkers must be applicable especially to widely used cell types that are easily derived from accessible human tissues such as blood and skin.

Such a potential biomarker that has gained significant interest in recent years is DNA methylation (DNAm). Chronological time has been shown to elicit predictable hypo- and hyper-methylation changes at many regions across the genome [1–5], and as a result, DNAm based biomarkers of aging were developed to estimate chronological age [6–10]. The blood-based age estimator by Hannum (2013) [9] and the pan-tissue estimator by Horvath (2013) [6] produce age estimates (DNAm age) that are widely used in epidemiological studies [11,12]. Mathematical adjustment of these age estimates in context of their corresponding chronological ages produces a measure of the rate of epigenetic aging, which is referred to as epigenetic age acceleration that can take a positive or negative value. Positive values of epigenetic age acceleration (indicative of faster epigenetic aging) have been repeatedly observed to be associated with many age-related diseases and conditions [11–24]. This indicates that epigenetic age is more than an alternative measure of chronological age but is instead an indicator of the state of health and as such, of biological age.

As indicated by its name, the pan-tissue age estimator applies to all sources of DNA (except for sperm) [6]. Despite its many successful applications, the pan-tissue DNAm age estimator performs sub-optimally when used to estimate fibroblast age [6]. This is particularly perplexing because fibroblasts are widely used in *ex vivo* studies of various interventions. As a case in point, the Progeria Research Foundation provides fibroblast lines derived from skin biopsies from patients with Hutchinson Gilford Progeria Syndrome (HGPS) for use in research. In spite of clear acceleration of clinical manifestations of aging in HGPS, this is not mirrored in epigenetic age measurements by current DNA methylation-based estimators [6]. While this could be due to a genuinely interesting distinction between epigenetic and phenotypic aging, it is also possible that the current epigenetic age estimators fail to capture aspects of aging that are specific to fibroblasts and epithelial cells. The discernment between the two possibilities requires an age estimator that is well-suited for accurately measuring the epigenetic age of fibroblasts. However, an epigenetic age estimator that is highly accurate and equally compatible with fibroblasts and other readily accessible human cells is currently not available. Such an epigenetic age estimator would be very valuable in performing ex vivo experiments because it would allow testing anti-aging properties of new compounds in human cells and minimize the need to carry out such tests in humans. Ex vivo studies often employ keratinocytes, fibroblasts and microvascular endothelial cells, which can be readily isolated from skin biopsies.

Here, we describe a novel powerful epigenetic age estimator (called the skin & blood clock) that outperforms existing DNAm-based biomarkers when it comes to estimating the chronological ages of human donors of fibroblasts, keratinocytes, microvascular endothelial cells, skin cells, coronary artery endothelial cells, lymphoblastoid cells, blood, and saliva samples.

## RESULTS

### DNA methylation data sets

We analyzed both novel and existing DNA methylation data sets that were generated on the Illumina Infinium platform (Table 1). DNA was extracted from human fibroblasts, keratinocytes, buccal cells, endothelial cells, blood, and saliva. We analyzed data from two Illumina platforms (Infinium 450K and the EPIC array, also known as the 850K array) to ensure that the resulting estimator would apply to the latest Illumina platform (the EPIC array).

**Table 1:** DNA methylation data. The rows correspond to Illumina DNA methylation data sets.

**No.**	**Data Source**	**Use**	**n**	**Source**	**Median Age (Range)**
1	existing, Portales-Casamar 2016, GSE80261	Train	216	Buccal	11 (5,18)
2	existing, Berko 2014, GSE50759	Train	96	Buccal	7 (1,28)
3	novel, blood methylation	Train	278	whole blood	69 (2,92)
4	existing, Yang 2017, GSE104471	Train	72	Epithelium	30 (24,74)
5	existing, Ivanov 2016, GSE77136	Train	21	Fibroblast	33 (0.1,85)
6	existing, Wagner 2014, GSE52026	Train	10	Fibroblast	37 (23,63)
7	novel fibroblasts	Train	48	Fibroblast	50 (0.42,94)
8	novel, Cell Applications	Train	11	Fibroblast	56 (7,94)
9	existing, Borman 2016, SkinE-MTAB-4385	Train	108	Skin	49.25 (18,78)
10	existing, cord blood, GSE79056	Train	36	cord blood	0 (-0.28,0.04)
11	existing, Jessen 2016, GSE94876	Test	120	Buccal	46 (35,60)
12	Lussier 2018, GSE109042	Test	53	Buccal	10 (3.5,18)
13	existing, Vandiver 2015, GSE51954	Test	78	Dermis+Epidermis	65 (20,92)
14	novel, Kenneth Raj	Test	23	Endothelial	19 (19,19)
15	novel, Kenneth Raj	Test	44	Endothelial	19 (17,26)
16	novel, Kenneth Raj	Test	48	Fibroblast	0 (0,0)
17	novel, Kenneth Raj	Test	48	Fibroblast	0 (0,0)
18	novel, Progeria Research Foundation+ vendors	Test	88	Fibroblast	8 (0,77)
19	novel, Junko Oshima	Test	11	Fibroblast	36 (0,62)
20	novel, Kenneth Raj	Test	43	Keratinocyte	0 (0,0)
21	novel, Blood methylation Inf 450	Test	100	Whole Blood	53 (19,82)
22	novel, Lymphoblastoid cell	Test	100	Lymphoblast	53 (19,82)
23	novel, Saliva methylation	Test	120	Saliva	44 (18, 81)
24	existing, Horvath 2015, GSE111223	Test	229	Saliva	68 (36,88)
25	existing, cord blood, GSE62924	Test	38	cord blood	0 (-.10,0.04)
26	existing, cord blood, GSE80283	Test	183	cord blood	-0.22(-0.3,-0.1)

The table reports the data set number, relevant citation (first author and publication year), public availability (for example, Gene Expression Omnibus identifier), sample size (n), source of the DNA (for example, tissue), median age, age range (minimum and maximum age). The column ‘Use’ reports whether the data set was used as a training set or test set.

### The DNAm age estimator for skin and blood

To ensure an unbiased validation of the test data, we used only the training data to define and construct the DNAm age estimator. As detailed in Methods, a transformed version of chronological age was regressed on methylation states of CpGs using elastic net regression [25], which automatically selected 391 CpGs (Methods). We refer to the 391 CpGs as (epigenetic) clock CpGs since their weighted average (formed by the regression coefficients) amounts to a highly accurate epigenetic aging clock.

**Figure 1:**
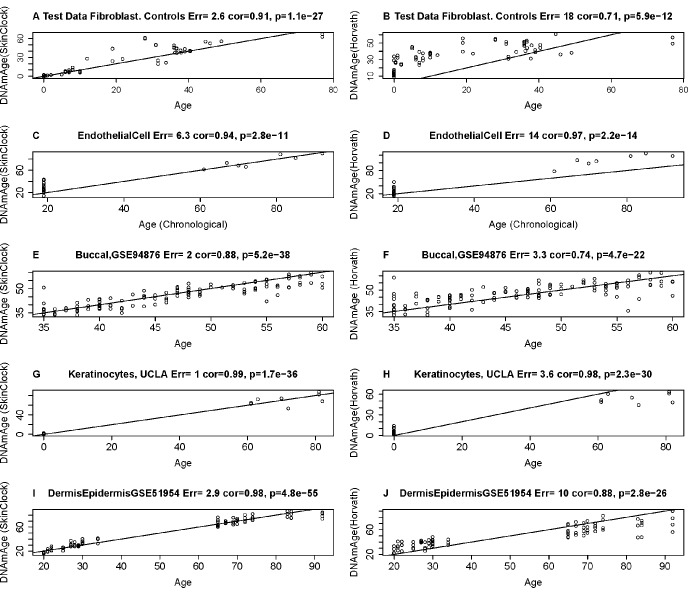
**Age estimation accuracy of the skin & blood clock in fibroblasts, keratinocytes, and microvascular endothelial cells.** The left and right panels relate chronological age (x-axis) to DNAm Age estimates (y-axis) from the skin & blood clock (**A**,**C**,**E**,**G**,**I**) and the pan-tissue clock (Horvath 2013) (**B**,**D**,**F**,**H**,**J**) [6], respectively. Each row corresponds to a different tissue/cell type. DNA methylation data from fibroblasts (**A**,**B**), microvascular endothelial cells **C**,**D**), buccal epithelial cells (**E**,**F**), keratinocytes (**G**,**H**), and whole skin (dermis/epidermis) samples (**I**,**J**). Each panel reports the Pearson correlation coefficient and the error (defined as median absolute deviation between DNAm age and chronological age).

**Figure 2:**
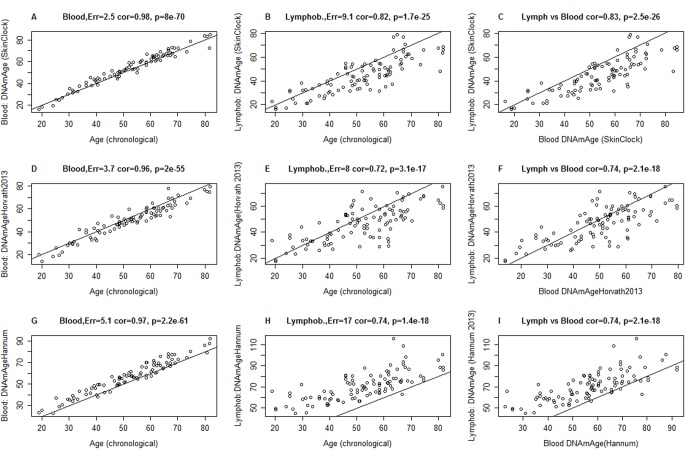
**Comparison of DNAm age estimators in whole blood and lymphoblastoid cell line data.** The rows correspond to 3 different age estimators: (**A**,**B**,**C**) the novel skin & blood clock (**D**,**E**,**F**), the pan-tissue clock (Horvath 2013) [6], (**G**,**H**,**I**) Hannum clock 9]. Panels in the first and second column report the accuracy in blood (**A**,**D**,**C**) and lymphoblastoid cell lines (**B**,**E**,**H**), respectively. Panels in the third column (**C**,**F**,**I**) report the relationship between DNAm age estimates in blood (x-axis) versus those in lymphoblastoid cell lines (y-axis). Panels report Pearson correlation coefficient and the estimation error, which is defined as median absolute deviation between the DNAm age estimate and chronological age. The lymphoblastoid cell lines were generated from the same individuals for whom whole blood was assessed, which facilitated the comparison in the third column.

The following description will demonstrate that the resulting age estimator (referred to as skin & blood clock) performs remarkably well across a wide spectrum of cells that are most frequently used in *ex vivo* studies. The new skin & blood clock even outperforms the pan-tissue clock (Horvath 2013) [6] in all metrics of accuracy (age correlation, median error) in fibroblasts, microvascular endothelial cells, buccal epithelial cells, keratinocytes, and dermis/epidermis samples (Figure 1 and Supplementary Figure 1). As indicated by its name, the new skin & blood clock is also a highly accurate age estimator of blood methylation data, where it provides more accurate age estimates than the widely used estimators by Horvath (2013) [6] and Hannum (2013) [9] (Figure 2A,D,G and Supplementary Figure 2). Further, it outperforms the Horvath and Hannum DNAm age estimators when applied to lymphoblastoid cell lines (Figure 2B,E,H), i.e. B cells that have been immortalized using EBV transformation. Interestingly, the DNAm age of blood is highly correlated with the DNAm age estimate of the lymphoblastoid cell line collected from the same donor at the same time (r=0.83, Figure 2C). The skin & blood clock accurately estimates age in two different saliva DNA methylation data sets (age correlations r=0.9 and r=0.95) and outperforms the pan-tissue DNAm age estimator in these data (Supplementary Figure 3). The skin & blood clock also applies to cord blood samples as can be seen from the fact that it accurately estimates gestational age in three different data (with correlations ranging from r=0.15 to r=0.66, Supplementary Figure 4).

### Skin & blood clock applied to brain, liver, bone, and other body parts

The skin & blood clock leads to DNAm age estimates that strongly correlate with chronological age in a host of different cell types and tissues including sorted neurons and glial cells (Supplementary Figure 5), brain samples (Supplementary Figure 6), liver samples (Supplementary Figure 7), and trabecular bone samples (Supplementary Figure 8). In the following, we provide more details on the individual studies.

In sorted neurons, DNAm age correlates strongly (r=0.83) with chronological age but the DNAm age estimator is substantially lower than chronological age (mean DNAm Age=6 years in a group of people whose mean chronological age is 31 years, Supplementary Figure 5A). Interestingly, the DNAm age of glial cells is significantly higher than that of neurons from the same individual (p=0.00024, Supplementary Figure 5B,C). Chronological age is also highly correlated with DNAm age estimates in different brain regions (r between 0.67 and 0.96, Supplementary Figure 6) but the age estimates tend to be systematically lower than chronological age. In particular, the cerebellum ages substantially slower than other brain regions echoing previous results for the pan-tissue clock [17].

DNAm age of liver tissue is highly correlated with chronological age (r=0.86, Supplementary Figure 7) and the corresponding measure of epigenetic age acceleration correlates with body mass index (r=0.27, p=0.027, Supplementary Figure 7B). These results echo earlier results obtained from the pan-tissue DNAm age estimator [13].

An analysis of 30 different body parts from a 112 year old woman reveals a) that most body parts have roughly the same age and b) that the cerebellum is substantially younger (Supplementary Figure 9) which is consistent with previous results [17].

### Epigenetic age of fibroblasts from Hutchinson Gilford Progeria fibroblasts

**Figure 3:**
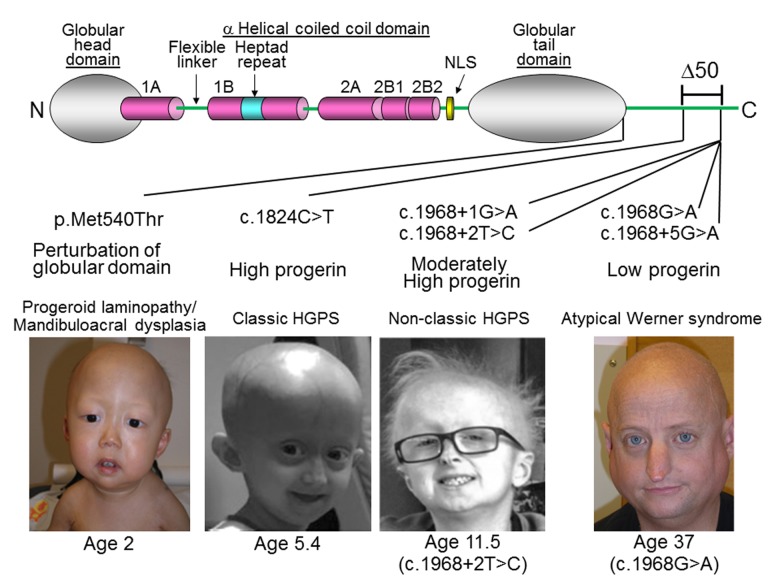
***LMNA* mutations in progeria patients. The diagram shows the structure of lamin A.** It consists of globular head domain, linker regions, α-helical coiled coil domain and globular tail domain. Locations of the progeria *LMNA* mutations in this study were shown with molecular mechanism of mutant lamin A protein and clinical phenotype, as previously reported in [34] (p.Met540Thr) [29], (c.1824C>T) [30], (c.1968+1G>A) [31], (c.1968+2T>C), and [36] (c.2968G>A and c.1968+5G>A). Δ50 indicates the region of deletion in progerin, also present in ZMPSTE24 mutant progeria [32]. Photos were reproduced with permission.

Previously, the use of the pan-tissue clock revealed epigenetic age acceleration in segmental progeroid syndromes such as Down syndrome and Werner syndrome [15,24], but not in syndrome X, whose patients exhibit dramatically delayed development (seemingly eternal toddler-like state) [26]. The status of the epigenetic aging rate in regards to HGPS and Atypical Werner Syndrome (AWS) is less clear. These two conditions can be caused by different progeroid mutations of the *LMNA* gene (Figure 3). It is not yet known whether HGPS patients, who generally appear normal at birth but exhibit a “failure-to-thrive” syndrome, exhibit any epigenetic age acceleration. HGPS is associated with many clinical manifestations of accelerated aging including loss of subcutaneous fat, joint contractures, and a striking aged appearance during the first to third years of life [27]. Virtually all HGPS patients die of myocardial infarction at a median age of 14.6 years [28]. Classic HGPS is caused by a recurrent heterozygous pathological variant, c.1824C>T in exon 11 of the *LMNA* gene, which activates a cryptic splice site and causes a 50-amino acid in-frame deletion (Δ50) [29]. The resulting abnormal protein, termed progerin, lacks the proteolytic site for an essential but transient post-translational modification by the ZMPSTE24 metalloprotease. This causes retention of the C-terminal farnesylated moiety, resulting in aberrant nuclear structure and function [29]. Non-classical HGPS mutations at the exon 11 and intron 11 boundary, including c.1968+1G>A [30] and c.1968+2T>C [31], can also activate the cryptic splice site, leading to the accumulation of progerin and an infantile-onset HGPS phenotype. Biallelic ZMPSTE24 mutations also cause accumulations of farnesylated lamin A and a varying degree of progeroid phenotypes, depending on the residual enzymatic activity of ZMPSTE24 [32,33]. In rare instances, a homozygous amino acid substitution of lamin A can present with a phenotype similar to HGPS or mandibuloacral dysplasia, as described in cases with [p.Met540Thr; p.Met540Thr] [34] and [p.Thr528Met; p.Met540Thr] [35].

A small subset of cases of Atypical Werner syndrome (AWS) (those with some features of Werner syndrome, without mutations in *WRN* or altered expressions of the WRN protein) may be caused by accumulations of low levels of progerin [36,37]. Pathological lamin A variants found in some patients with AWS include c.1968G>A and c.1968+5G>A [36]. While there is a general genotype-phenotype correlation between the amount of progerin and the severity of the disease, the amounts and structures of progerin can vary among those who carry the same *LMNA* splice mutation, and the severity of the disease can vary among patients within the same family [36,37]. The median age of death of classic HGPS is ~14.6 years [38], while the range in AWS patients with low levels of progerin is 37 to 60 years [36].

**Figure 4:**
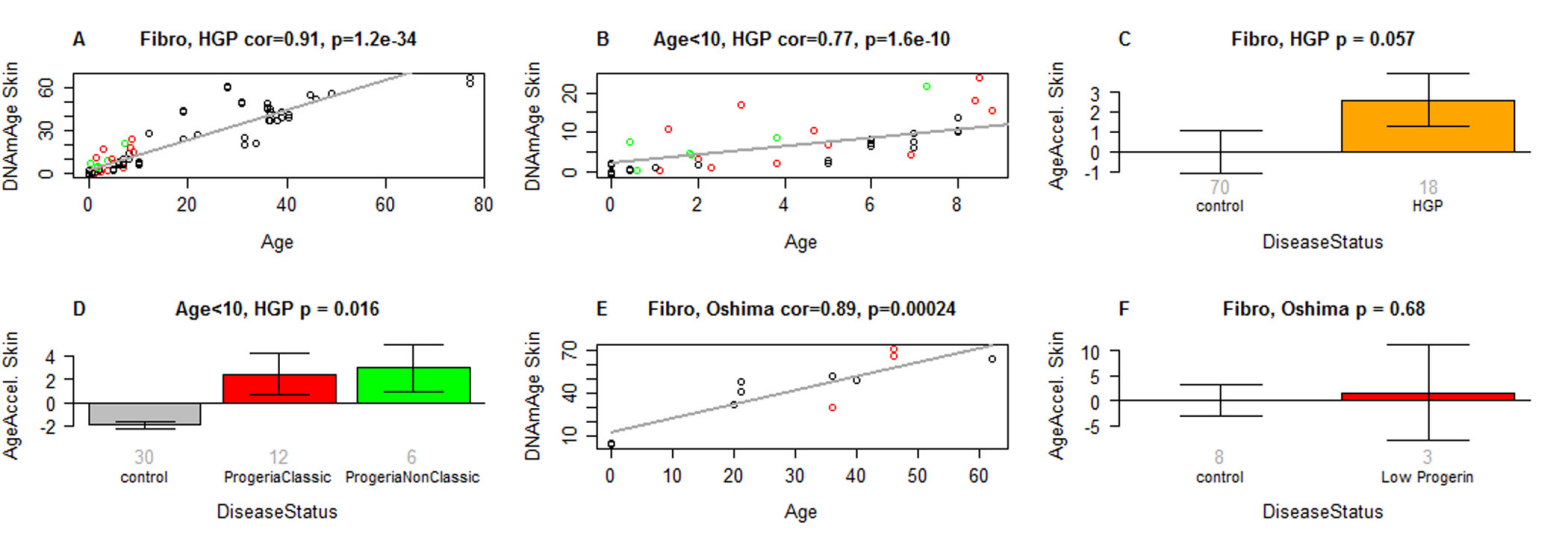
**Skin & blood clock analysis of fibroblasts from HGP individuals of the Progeria Research Foundation**. (**A**,**B**) The new skin & blood clock was used to estimate DNAm age (y-axis) in fibroblasts from HGP individuals and controls. (**A**) All individuals. (**B**) Children younger than 10 years old. Dots are colored by disease status: red=classical progeria, green=non-classical progeria, black=controls. The grey line corresponds to a regression line through control individuals. The epigenetic age acceleration effect for each individual (point) corresponds to the vertical distance to the black regression line. The fact that red and green points tend to lie above the grey line indicates that HGP cases exhibit suggestive accelerated epigenetic aging effect. (**C**) Mean epigenetic age acceleration (y-axis) versus HGP status. By definition, the mean age acceleration measure in controls is zero. (**D**) Epigenetic age acceleration (y-axis) versus disease status in individuals younger than 10. (**E**, **F**) report results for fibroblast samples from atypical Werner syndrome cases (low progerin) provided by co-author Junko Oshima. (**E**) DNAm age versus chronological age for atypical Werner syndrome samples (colored in red) and controls (colored in black). (**F**) Epigenetic age acceleration versus disease status. The title of the bar plots also reports a P-value from a nonparametric group comparison test (Kruskal Wallis test). Each bar plot reports the mean value and one standard error.

The original pan-tissue DNAm age estimator did not detect any age acceleration in HGPS individuals (Supplementary Table 1). By contrast, the application of the novel skin & blood clock showed that while DNAm age is highly correlated with chronological age in normal fibroblasts, those from HGPS cases exhibited accelerated epigenetic aging (Figure 4). The correlation between age and DNAm age in HGPS children (<10 years old) is substantially lower (r=0.71) than that of control children (r=0.71, Figure 4B). The epigenetic age acceleration effects become particularly pronounced after adjusting for differences in cell population doubling levels and when the analysis was restricted to children who are younger than 10 years old (p=0.00021, Table 2, Table 3). There is a non-significant trend of increased DNAm age in Atypical Werner Syndrome cases with low levels of progerin. It is perhaps not unexpected that AWS, which presents with a lower progerin concentration (Figure 3) is not significantly associated with greater magnitude of epigenetic age acceleration.

**Table 2:** Epigenetic clock results for fibroblast samples from The Progeria Research Foundation.

**Cell-line ID**	**Progeria**	**Mutation**	**Sex**	**Age**	**DNAmAgeSkinClock**	**AgeAccelSkinClock**
PSADFN086	NonClassic	LM Exon 11 c.1968+1G>A	m	0.58	0.39	-3.49
PSADFN257	NonClassic	LM Exon 10 homozygous c.1619 T>C (p.Met540Thr)	m	1.83	4.44	-0.51
PSADFN257.replicate	NonClassic	LM Exon 10 homozygous c.1619 T>C (p.Met540Thr)	m	1.8	4.84	-0.08
PSADFN317	NonClassic	ZMPste24 Exon 6 heterozygous c.743C>T(p.Pro248Leu)and Exon 10 heterozygous c.1349G>A (p.Trp450Stop)	m	3.8	8.86	2.23
PSADFN318	NonClassic	ZMPste24 Exon 6 heterozygous c.743 C>T(p.Pro248Leu)and Exon 10 heterozygous c.1349G>A (p.Trp450Stop)	m	0.4	7.48	3.75
PSADFN392	NonClassic	LM Exon 11 c.1968+2T>C	m	7.3	21.61	11.99
HGADFN003	Classic	LM Exon 11 heterozygous c.1824C>T	m	2	3.39	-1.70
HGADFN169	Classic	LM Exon 11 heterozygous c.1824C>T	m	8.5	23.73	13.08
HGADFN143	Classic	LM Exon 11 heterozygous c.1824C>T	m	8.8	15.61	4.71
HGADFN167	Classic	LM Exon 11 heterozygous c.1824C>T	m	8.4	17.88	7.32
HGADFN271	Classic	LM Exon 11 heterozygous c.1824C>T	m	1.3	10.73	6.24
HGADFN164	Classic	LM Exon 11 heterozygous c.1824C>T	f	4.66	10.64	3.28
HGADFN178	Classic	LM Exon 11 heterozygous c.1824C>T	f	6.92	4.36	-4.93
HGADFN122	Classic	LM Exon 11 heterozygous c.1824C>T	f	5	6.96	-0.70
HGADFN127	Classic	LM Exon 11 heterozygous c.1824C>T	f	3.8	2.10	-4.53
HGADFN155	Classic	LM Exon 11 heterozygous c.1824C>T	f	1.1	0.59	-3.73
HGADFN188	Classic	LM Exon 11 heterozygous c.1824C>T	f	2.3	1.23	-4.11
HGADFN367	Classic	LM Exon 11 heterozygous c.1824C>T	f	3	17.10	11.16

The columns report the cell line identifier, the disease status, mutational analysis, sex, age, DNAm age estimate (based on the skin & blood clock), and the measure of age acceleration (defined as residual from a regression line). Classic HGPS cases exhibit the following mutation: LMNA Exon 11, heterozygous c.1824C>T (p.Gly608Gly). By contrast, non-classic HGPS cases exhibit mutations elsewhere in the LMNA gene.

**Table 3:** Multivariate regression model analysis of HGPS based on the novel skin & blood clock.

**Outcome: DNAmAge (SkinClock)**						
	**Data: All, n=88**			**Data: Age<10, n=44**		
**Covariate**	**Coef**	**St. Error**	**P-value**	**Estimate**	**SE**	**P-value**
**Intercept**	-3.55	2.99	2.39E-1	7.34	2.97	1.84E-2
**Age**	1.64	1.29E-1	3.44E-20	-5.90E-1	8.33E-1	4.84E-1
**Age^2**	-1.07E-2	2.08E-3	2.14E-6	2.40E-1	9.58E-2	1.70E-2
**Fibroblast Population Doubl. Level**	4.46E-1	1.65E-1	8.52E-3	-1.20E-1	1.32E-1	3.71E-1
**HGP.Disease**	4.81	2.27	3.76E-2	5.18	1.25	2.12E-4

DNAm age is regressed on chronological age, the square of age, the population doubling level of the fibroblast cell culture, and HGPS disease status. The table reports estimate of the regression coefficients and corresponding standard errors, Wald test P-values. The left panel and right panel report the results for all n=88 fibroblast samples and for n=44 samples from children (younger than 10 years old), respectively.

Although non-classic HGPS patients often present at later ages, they can nevertheless be diagnosed at ages that are slightly younger than patients with classic HGPS [27]. It should indeed be noted that the cases examined in this study (see Methods for mutation details), have exceptionally early manifestations – as early as birth or younger than 5 months of age. Interestingly, their DNA methylation age acceleration is comparable and consistent with that of classic HGPS, which as mentioned, is an early onset progeria condition (Figure 4D).

Detailed results for the lines of skin fibroblasts provided by The Progeria Research Foundation are presented in Table 2 and Supplementary Table 2. The skin & blood clock provides marginally significant evidence (p=0.062) that fibroblasts from boys with classic HGPS are epigenetically older than those from girls with classic HGPS, but this gender effect is not apparent when classic and non-classic HGPS samples are pooled for analyses (Supplementary Figure 10).

It is to be further noted that the epigenetic age acceleration of HGPS fibroblasts revealed by the skin & blood clock escaped detection when measurements were carried out with the pan-tissue clock; indeed, the opposite appears to be the case (Supplementary Figure 11C, Supplementary Table 2). Evidently, the ability to detect such epigenetic age changes in fibroblasts is dependent upon the choice of the DNAm age estimator that is used.

### *Ex vivo* studies of anti-aging interventions

**Figure 5:**
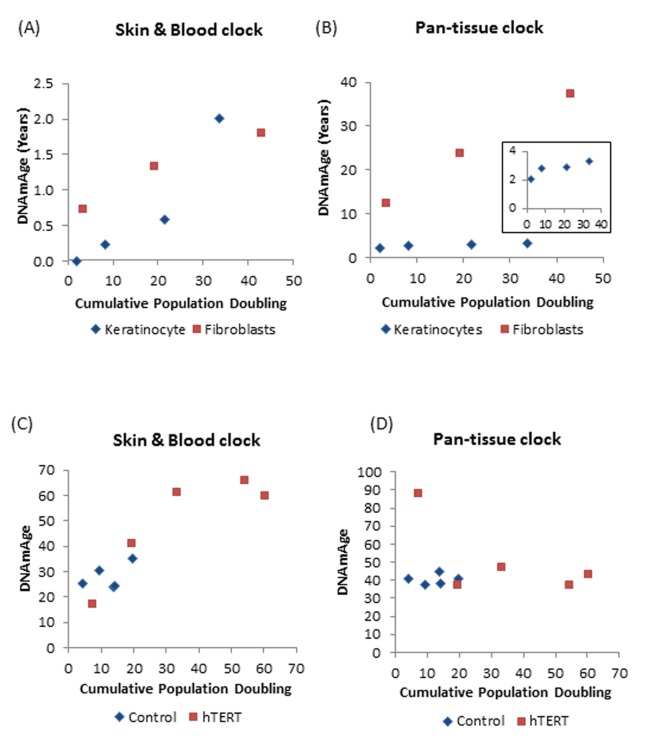
**DNAm age versus population doubling levels**. Each panel reports a DNAm age estimate (y-axis) versus cumulative population doubling level, respectively. Plots in the left and right panels correspond to the new skin & blood clock (**A**,**C**) and the pan-tissue clock (**B**,**D**) respectively. (**A**,**B**) Tracking of the epigenetic ages of neonatal fibroblasts (Red squares) and keratinocytes (Blue diamonds) in function of population doubling. Inset graph in (**B**) is a plot of ages of only the keratinocyte population (**C**,**D**). Epigenetic ages of human coronary artery endothelial cells derived from a 26 year old donor, in function of cumulative population doubling. Ages of uninfected control cells, which senesced after cumulative population doubling of 20, are shown in blue while those bearing hTERT, with extended proliferative capacity are in red. The blue dots with the highest cumulative doubling are at points when the cells reached replicative senescence. Cells with hTERT (represented by red squares) do not senesce and the last dots indicate the termination of the experiment.

While it may appear obvious that the skin & blood clock is superior in terms of compatibility with fibroblasts, it was still necessary to verify and validate this deduction by applying this clock to non-progeria fibroblasts and other cell types. To this end, fibroblasts derived from non-progeria neonatal foreskins are ideal as they pose minimal to no confounding factors that could alter their age. While the skin & blood clock correctly estimated the neonatal fibroblast cells to be of ages close to zero years, the pan-tissue age estimator leads to age estimates that are greater than 10 years (Figure 5A and B). Analyses of other skin cell types namely, keratinocytes and microvascular endothelial cells derived from neonatal foreskins also demonstrated greater accuracy of the skin & blood clock over the other age estimators. This conclusion continues to hold true even when the analyses were extended to isogenic skin cells derived from adult tissues (Supplementary Figure 1).

Having established the robustness of the skin & blood clock in measuring age of cells isolated from human tissues, we proceeded to test the applicability of the clock on human cells cultured *ex vivo*. As observed previously using the pan-tissue age estimator, the skin & blood clock revealed that human fibroblasts cultured *ex vivo* undergo epigenetic aging. However, unlike the former which over-estimates the DNAm age of fibroblasts (Figure 5B), the skin & blood clock correctly estimated the age of the neonatal cells to within 6 months (Figure 5A). Proliferation of human fibroblasts in culture, measured as population doubling, was observed to correlate with continual increase in DNAm age. Similar correlation was also seen with neonatal foreskin keratinocytes (Figure 5A and B). Although this association was revealed by both clocks, the resolution and accuracy of the skin & blood clock are clearly much greater and better. The new clock also out-performs the pan-tissue clock when applied to non-blood or skin cells; namely, the human coronary artery endothelial cells, whose increase in epigenetic age in function of population doubling was readily detected by the former but not the latter. Cells whose proliferative capacity was extended by hTERT beyond their otherwise natural limits (senescence), continued to age epigenetically, underscoring the correlation between cellular proliferation and DNA methylation aging.

By its ability to quantitatively track aging of human cells *ex vivo,* the skin & blood clock lends itself to be used in the development of an *ex vivo* human cell aging assay that can be used for testing and screening compounds with anti-aging or pro-aging effects.

### Analysis of blood samples from human cohort studies

Epigenetic clocks are associated with a host of different age-related phenotypes and conditions (reviewed in [11,12]). In the following, we report the results of several post-hoc analyses that demonstrate that the new skin & blood clock satisfies most of the properties observed for other blood based DNAm age estimators.

Similar to what has been observed with previous age estimators, epigenetic age acceleration in blood (according to the skin & blood clock) is highly predictive of time to all-cause mortality (p=9.6E-7) according to a univariate Cox regression model fixed effects meta-analysis across multiple epidemiological cohort studies (Supplementary Figure 12).

Blood samples from individuals with Down syndrome exhibit positive epigenetic age acceleration compared to controls (p=0.034, Supplementary Figure 13) consistent with previous findings [15].

Similar to the previous epigenetic aging clock analyses of blood [21], cross sectional studies of n=3700 blood samples from postmenopausal women from the Women's Health Initiative revealed relationships to lifestyle factors and dietary variables (Supplementary Table 3). Slow epigenetic age acceleration in blood was associated with higher education (p=6E-5), physical exercise (p=4E-3), fish consumption (p=2E-4), poultry consumption (p=3E-4), high mean carotenoid levels (p=8E-6), beta cryptoxhanthin (p=2E-7), beta carotene levels (p=4E-4), and HDL levels (p=5E-4, Supplementary Table 3). Conversely, faster epigenetic aging in blood is associated with higher C-reactive protein levels (p=1E-3), body mass index (p=0.01), triglyceride levels (p=3E-3), and insulin (p=2E-3). However, it is worth emphasizing that the respective correlation coefficients were weak (|r|<0.11, Supplementary Table 3). Physical exercise was associated with a slower epigenetic aging effect in African American women (p=4E-3) and perhaps in Caucasian women (p=0.07) but not in Hispanic women (p=0.74). Current smoking status was only associated with increased age acceleration in Caucasian women (p=0.04).

Epigenetic age acceleration is highly conserved across a 9 year follow up time period (r=0.71, Supplementary Figure 14). In other words, if an individual exhibits positive epigenetic age acceleration at age 40 then he/she will probably continue to exhibit positive epigenetic age acceleration at age 49.

Collectively these characteristics demonstrate that although the new clock is highly and uniquely accurate for skin cells, it has not acquired this at the cost of losing any of the features shared amongst existing age estimators in being also highly accurate with blood DNA methylation data, buccal cells, saliva. As such, this clock could become useful for forensic applications.

### Relationship to other DNAm age estimators

The skin & blood clock (based on 391 CpGs) shares 45 CpGs (out of 71 CpGs) with the blood-based clock from Hannum (2013) and 60 CpGs (out of 353 CpGs) with the pan tissue clock from Horvath (2013) as detailed in Supplementary Table 4. Despite this significant overlap, epigenetic age acceleration of the skin & blood clock exhibits only moderate correlations with corresponding epigenetic age acceleration measures by Horvath (2013) and Hannum (2013) (r=0.5 and r=0.59, p<1.E-110) in the blood samples from the Women's Health Initiative (BA23 study).

### Leukocyte telomere length and blood cell counts

We find a weak a weak negative correlation between leukocyte telomere length and epigenetic age acceleration of blood (r=-0.087, p=0.0088 in n=905 samples from the Framingham Heart Study, r =-0.081, p=0.0011 in n=1639 samples from the Jackson Heart Study, and r=-0.117, p=0.00079 in the 818 samples from the Women's Health Initiative). This weak negative relationship between telomere length and epigenetic age acceleration is similar to that of the Hannum-based DNAm age estimator and other blood based biomarkers [11,39,40].

Epigenetic age acceleration measured by the skin & blood clock is only weakly correlated with, or affected by blood cell type counts, as is evident from the analyses of postmenopausal women from the Women's Health Initiative (Supplementary Figure 15). The strongest correlations are observed with exhausted CD8+ T cells (r=0.22), naive CD8+ T cells (r=-0.21), and naive CD4+T cells (r=-0.19, Supplementary Figure 15B-D). These correlations suggest that individuals with positive epigenetic age accelerations exhibit an adaptive immune system that is older than expected.

## DISCUSSION

We present a new DNA methylation-based biomarker (based on 391 CpGs) that was developed to accurately measure the age of human fibroblasts, keratinocytes, buccal cells, endothelial cells, skin and blood samples. Perhaps unexpectedly, we also observe strong age correlations in sorted neurons, glia, brain, liver, and bone samples. The need for the new skin & blood clock became apparent when it was observed that the existing DNA methylation-based age estimators that are highly accurate in measuring ages of blood and many cell types of the body, perform poorly when applied to human fibroblasts and other skin cells. The implications of this anomaly extend beyond theoretical curiosity as it impacts the reliability of conclusions drawn from epigenetic age analyses of skin cells. As a case in point, the apparent lack of epigenetic age acceleration of HGPS fibroblasts indicated by measurements using the pan-tissue age estimator revealed an important limitation.

Skin cells are among the most common cell types employed in laboratories. This is owed largely to the ease by which cells such as keratinocytes, fibroblasts, and microvascular endothelial cells can be isolated from skin, allowing cells from many donors to be easily acquired and used; a characteristic that is not afforded by internal organs. The ability to use these cells to investigate epigenetic age *ex vivo* is paramount if we are to identify constituents of the epigenetic clock and elucidate how they function together to drive the ticking of the clock.

The skin & blood that we derived is well-suited to meet all these needs. By applying it to fibroblasts from HGPS cases, we detect a significant epigenetic age acceleration effect after adjusting for fibroblast population doubling levels. For reasons yet to be determined, the pan-tissue DNA methylation age estimator failed to detect this subtle increase in epigenetic age acceleration. In considering the modest increase in age acceleration of HGPS cells, it is worth noting that changes in the methylation state of clock CpGs in the early years of life already occur at a frenetic rate, which is approximately twenty-four times greater than that which takes place after the age of twenty [6]. Hence, it is difficult to envisage that the accelerated rate of epigenetic aging in HGPS cells from young donors could be very much greater in magnitude. This hypothesis can in theory be tested by measuring the epigenetic age of HGPS cells from patients older than twenty years of age, when the basal rate of normal epigenetic aging is significantly reduced, allowing for any age acceleration to become even more apparent. It is however difficult to achieve this as the median age of death of HGPS patients is approximately 14 years old. The ability of the skin & blood clock to nevertheless detect epigenetic age acceleration in young HGPS patients over and above an already very high normal background rate, attests to its sensitivity.

It is also conceivable that there may be specific, as well as overlapping aging mechanisms in patients with different segmental progeroid syndromes (i.e. HGPS versus classical WS) that differentially contribute to their respective rates of DNAm acceleration. Alternatively, these differences might be attributable, at least in part, to the consequences rather than the causes of the patterns of diverse pathologies that characterize these different phenotypes.

In addition to resolving the conundrum of HGPS described above, the skin & blood clock outperforms widely used existing biomarkers when it comes to accurately measuring the age of an individual based on DNA extracted from skin, dermis, epidermis, blood, saliva, buccal swabs, and endothelial cells. Thus, the biomarker can also be used for forensic and biomedical applications involving human specimens. The biomarker applies to the entire age span starting from newborns, e.g. DNAm of cord blood samples correlates with gestational week (Supplementary Figure 4). Furthermore, the skin & blood clock confirms the effect of lifestyle and demographic variables on epigenetic aging. Essentially it highlights a significant trend of accelerated epigenetic aging with sub-clinical indicators of poor health. Conversely, reduced aging rate is correlated with known health-improving features such as physical exercise, fish consumption, high carotenoid levels (Supplementary Table 3). As with the other age predictors, the skin & blood clock is also able to predict time to death. Collectively, these features show that while the skin & blood clock is clearly superior in its performance on skin cells, it crucially retained all the other features that are common to other existing age estimators.

The skin & blood clock is particularly well suited for forensic applications because it greatly outperforms the considered alternative DNAm based estimators when it comes to measuring chronological age in blood, buccal cells, saliva, and other cell types (Figure 1, Figure 2, Supplementary Figures 2-4).

The performance of the skin & blood clock is equally impressive when applied to *ex vivo* cell culture system. Studies with fibroblasts and endothelial cells revealed that increase in population doublings is significantly associated with increased DNAm age including hTERT immortalized cells, which corroborates the findings in previous studies [41,42].

We have coupled the skin & blood clock with human primary cell cultures to generate an *ex vivo* human cell aging assay that is highly sensitive. This assay is able to detect epigenetic aging of a few years, within a few months. The benefits of this assay are self-evident. The two most obvious are its use to test and screen for potential pharmaceuticals that can alter the rate of epigenetic aging, and its use to test and detect potential age-inducing hazards in the arena of health protection.

Many of our key observations are critically dependent upon the choice of a DNAm age estimator, i.e., they could only be observed with the new skin & blood clock assay. For example, the original pan-tissue clock could not detect an age acceleration effect in HGPS. Looking ahead, there might be valuable applications of this more robust epigenetic clock for example, in the evaluation of clinical trials of pharmaceutical interventions in segmental progeroid syndromes such as the most recent clinical trial of a farnesyltransferase inhibitor, lonafarnib, to treat HGPS that reportedly lowers mortality rates (6.3% death in the treated group vs 27% death in the matched untreated group after 2.2 years of follow-up) [28]. We are likely to see an increase of such clinical trials. For example, *in vitro* studies of the effects of rapamycin or another mTOR inhibitor, metformin, showed a reduction of progerin accumulation accompanied by the amelioration of cellular HGPS phenotypes [43,44]. Reactivation of the antioxidant NRF2 was also shown to alleviate cellular defects of HGPS in an animal model [45]. Beyond therapeutic aims, prophylactic interventions would certainly be sought-after, and the ability of the skin & blood clock to accurately measure the age of cells from highly accessible human tissues will reveal whether the tested treatments are widely targeted across cell and tissue types - an important feature that is not hitherto afforded by other age estimators.

Due to its superior accuracy, we expect that this novel set of epigenetic biomarkers will be useful for both *ex vivo* studies involving cultures of various somatic cell types, including fibroblasts, keratinocytes, and endothelial cells, as well as *in vivo* studies utilizing samples of peripheral blood and biopsies of skin.

## METHODS

The R software code underlying the new skin & blood clock can be found in the Supplement.

### Definition of DNAm age using a penalized regression model

A penalized regression model (implemented in the R package glmnet [46]) was used to regress a calibrated version of chronological age on the CpG probes in the training set. We restricted the analysis to CpGs that were present both on the Illumina 450K and EPIC platforms and were in one of the following subsets: 1) most significant CpGs with high positive/negative correlation with chronological age in different cell types or 2) 500 CpGs with the least significant correlation with age. The alpha parameter of glmnet was chosen as 0.5 (elastic net regression) and the lambda value was chosen using cross-validation on the training data. DNAm age was defined as predicted age.

### Fibroblasts from The Progeria Research Foundation

Human primary dermal fibroblast cell lines were obtained from The Progeria Research Foundation (PRF) Cell and Tissue Bank (www.progeriaresearch.org). The fibroblast cell lines originated from cases with classic mutations, non-classic mutations and parental controls as detailed in Table 2. The following citations provide additional details on cases carrying the specific variants: *LMNA* c.1968+1G>A heterozygote [30], *LMNA* c.1968+2T>C heterozygote [31], *LMNA* p.Met540Thr homozygotes [34] and compound heterozygotes of ZMPSTE24 p.Pro248Leu and p.Trp450* [32]. Additional details can be found in the Supplement.

### Isolation and culture of cells for *ex vivo* experiments

Informed consent was obtained prior to collection of human skin samples with approved by the Oxford Research Ethics Committee; reference 10/H0605/1. Human skin samples were acquired under ethical approval. Primary human skin keratinocytes, fibroblasts and microvascular endothelial cells were isolated from neonatal foreskin and adult facial/neck skin. Keratinocytes were cultured in CnT media (CellnTec) on fibronectin/collagen-coated plates, fibroblasts were cultured in DMEM (Sigma) supplemented with 10% foetal calf serum and CD31-selected microvascular endothelial cells were cultured in Endothelial Cell Growth Medium MV (PromoCell, C-22020) on gelatin-coated plates. Human Coronary Artery Endothelial Cells (HCAEC) from a male, aged 26 years, were obtained from Sigma and grown in MesoEndo Cell Growth Medium (Sigma). HCAEC were immortalized with pBABE-neo-hTERT (Addgene, cat. 1774) and after selection, were cultured in parallel with uninfected control until control reached senescence. All cells were maintained in a 37 °C, 5% CO2 humidified environment. At each passaging step, cells were counted, population doubling calculated and 10,000 were seeded into a fresh 10cm plate. Remaining cells were used for DNA extraction. Population doubling was calculated with the following formula: [log(number of cells harvested) – log(number of cells seeded)] x 3.32. Cumulative population doubling was obtained by addition of population doubling of each passage. Additional details can be found in the Supplement.

### Sample preparation

DNA was extracted from cells using the Zymo Quick DNA mini-prep plus kit (D4069) according to the manufacturer’s instructions and DNA methylation levels were measured on Illumina 450 or Illumina 850 EPIC arrays according to the manufacturer’s instructions.

### Blood methylation data from different epidemiological cohorts

A number of validation studies were used to test associations between DNAm age and various aging-related traits including time to all-cause mortality. Details on these studies can be found in the Supplement and in [20,40].

### Competing interests

The Regents of the University of California is the sole owner of a provisional patent application directed at this invention for which SH is a named inventor.

### Ethics approval

This study was reviewed by the UCLA institutional review board (IRB#14-000140, IRB#15-001479). Collection of human skin samples were carried out under the ethical approval issues by the Oxford Research Ethics Committee; reference 10/H0605/1 (United Kingdom)

## 

The views expressed are those of the authors and not necessarily those of the funding agencies or PHE.

## Supplementary Material

Supplementary Results, Methods and References

Supplementary Figures

Supplementary Tables

Supplementary Dataset 1

Supplementary Dataset 2
