# Supplementary material for: Epigenetic clock for skin and blood cells applied to Hutchinson Gilford Progeria Syndrome and ex vivo studies
Source: Aging (Albany NY). 2018 Jul 26;10(7):1758–75. doi: 10.18632/aging.101508 (PMC6075434; doi:10.18632/aging.101508)
Supplement: Supplementary Results, Methods and References [file aging-10-101508-s001.pdf]

SUPPLEMENTARY RESULTS, METHODS and REFERENCES

Contents

Fibroblasts from The Progeria Research Foundation 1
Isolation and culture of cells for *ex vivo* experiments 2
Normalization of DNA methylation data 2
Estimation of blood cell counts based on DNAm levels 2
Blood methylation data from large epidemiological cohorts 2
Statistical Methods 4
R software code 4

Fibroblasts from The Progeria Research Foundation

Human primary dermal fibroblast cell lines were obtained from The Progeria Research Foundation (PRF) Cell and Tissue Bank (www.progeriaresearch.org). The fibroblast cell lines originated from cases with classic mutations, non-classic mutations and parental controls as detailed in Table 2. The following citations provide additional details on cases carrying the specific variants: *LMNA* c.1968+1G>A heterozygote[1], *LMNA* c.1968+2T>C heterozygote[2], *LMNA* p.Met540Thr homozygotes [3] and compound heterozygotes of ZMPSTE24 p.Pro248Leu and p.Trp450* [4]. As detailed in Table 2, we generated DNA methylation data from the following cell lines that are described on the PRF webpage (https://www.progeriaresearch.org/):

PSADFN086, PSADFN257, PSADFN317, PSADFN318, PSADFN392, HGADFN003, HGADFN169, HGADFN143, HGADFN167, HGADFN271, HGADFN164, HGADFN178, HGADFN122, HGADFN127, HGADFN155, HGADFN188, HGADFN367, HGFDFN369, PRF319P8, PSFDFN319, PSFDFN327, PSFDFN394, PSFDFN319, HGMDFN090, HGMDFN368, PSMDFN320, HGMDFN368, PSMDFN320, PSMDFN326, PSMDFN346, PSMDFN393, HGFDFNDNA168.

Control samples

To avoid batch effect in the DNA methylation data, we generated control fibroblast samples for concurrent assays with fibroblasts from patients with HGPS. The control fibroblasts have been described in [5]. Fibroblast lines ranging in age from three days to 96 years were obtained from the NIA Aging Cell Repository at the Coriell Institute for Medical Research. The Coriell ID designations were:, RRID#: AG08498, RRID:CVCL_1Y51, AG07095, RRID:CVCL_0N66, AG11732, RRID:CVCL_2E35, AG04060, RRID:CVCL_2A45, AG04148, RRID:CVCL_2A55, AG04349, RRID:CVCL_2A62, AG04379, RRID:CVCL_2A72, AG04056, RRID:CVCL_2A43, AG04356, RRID:CVCL_2A69, AG04057, RRID:CVCL_2A44, AG04055, RRID:CVCL_2A42, AG13349, RRID:CVCL_2G05, AG13129, RRID:CVCL_2F55, AG12788, RRID:CVCL_L632, AG07725, RRID:CVCL_2C46, AG04064, RRID:CVCL_L624, AG04059, RRID:CVCL_L623, AG09602, RRID:CVCL_L607, AG16409, RRID:CVCL_V978, AG06234, RRID:CVCL_2B66, AG04062, RRID:CVCL_2A47, AG08433, RRID:CVCL_L625, AG16409, RRID:CVCL_V978, GM00302, RRID:CVCL_7277, AG01518, RRID:CVCL_F696, AG06234, RRID:CVCL_2B66.

Mycoplasma contamination is routinely ruled out for all cell cultures using LINE and PCR-based techniques. None of the cell lines we have used are among those listed the International Cell Line Authentication Committee (ICLAC) as commonly misidentified cell lines. Fibroblast cell lines were cultured and expanded in DMEM media (high glucose, Invitrogen) supplemented with 10% or 15% fetal bovine serum (Gibco), sodium pyruvate, non-essential amino acids, GlutaMAX (Invitrogen), Pen/Strep solution, and Beta-mercapto-ethanol. Fibroblast cell lines were expanded to a population doubling level (PDL) of ~19–21. The formula used to calculate PDL was PDL = 3.32*log (cells harvested/cells seeded) + previous PDL. Cell aliquots of early passages of all cell lines were kept frozen at −150°C in the above culture medium with additional 40% FBS and 10% DMSO.

Isolation and culture of cells for *ex vivo* experiments

Informed consent was obtained prior to collection of human skin samples with approval from the Oxford Research Ethics Committee; reference 10/H0605/1. Primary human skin keratinocytes, fibroblasts and microvascular endothelial cells were isolated from neonatal foreskin and adult facial/neck skin. The tissue was cut into small pieces and digested overnight at 4 °C with 0.5 mg/ml Liberase DH in CnT-07 keratinocyte medium (CellnTech) supplemented with 2x penicillin/ streptomycin (Sigma) and 2x gentamycin/amphotericin (Life Tech). Following digestion, the epidermis was peeled from the dermal layer, a single-cell suspension obtained by mechanical dissociation in trypsin-EDTA and seeded on collagen/fibronectin coated plates in CnT-07 medium. To isolate fibroblasts, dermal pieces were grown from explants in DMEM supplemented with 10% FBS. The remaining dermal tissue was digested in 2.5 mg/ml collagenase in HBSS (with calcium and magnesium) at 37 °C with frequent agitation for 1 h, passed through a 70 μm cell strainer and selected using CD31 magnetic Dynabead positive selection (Life Technologies, 11155D). Selected microvascular endothelial cells were then seeded on a gelatin-coated flask in Endothelial Cell Growth Medium MV (PromoCell, C-22020). All cells were maintained in a 37 °C, 5% CO2 humidified environment.

Normalization of DNA methylation data

The Illumina BeadChips measures bisulfite-conversion-based, single-CpG resolution DNAm levels at different CpG sites in the human genome. These data were generated by following the standard protocol of Illumina methylation assays, which quantifies methyltion levels by the β value using the ratio of intensities between methylated and un-methylated alleles. Specifically, the β value is calculated from the intensity of the methylated (M corresponding to signal A) and un-methylated (U corresponding to signal B) alleles, as the ratio of fluorescent signals $\beta = Max(M,0)/[Max(M,0)+Max(U,0)+100]$. Thus, β values range from 0 (completely un-methylated) to 1 (completely methylated). For WHI we used background corrected beta values, while InCHIANTI and the JHS data were normalized using the NOOB method [6].

Estimation of blood cell counts based on DNAm levels

We estimated blood cell counts using two different software tools. First, Houseman's estimation method [7] was used to estimate the proportions of CD8+ T cells, CD4+ T, natural killer, B cells, and granulocytes (mainly neutrophils). Second, the Horvath blood cell estimation method, implemented in the advanced analysis option of the epigenetic clock software [8, 9], was used to estimate the percentage of exhausted CD8+ T cells (defined as CD28-CD45RA-), the number (count) of naïve CD8+ T cells (defined as CD45RA+CCR7+) and plasma blasts cells. We and others have shown that the estimated blood cell counts have moderately high correlations with corresponding flow cytometric measures [7, 10].

Blood methylation data from large epidemiological cohorts

Women's Health Initiative (WHI), Framingham Heart Study, Jackson Heart Study, and InCHIANTI were used for evaluating the predictive accuracy for mortality and morbidity analyses. All but one epidemiological cohort used the Illumina Infinium 450K platform. However, the data from the Jackson Heart Study were generated on the EPIC array.

InCHIANTI included longitudinal (two time-points—1998 and 2007) phenotypic and DNAm data on n=456 male and female participants, ages 21-91 in 1998, and 30-100 in 2007.

Women's Health Initiative

Two separate subsamples were aggregated for our study within the WHI (BA23 and AS315). Both had baseline blood specimens collected after an overnight fast in EDTA tubes and stored at -70C. These samples were processed at the WHI core laboratory and select nutrient and cardiovascular biomarkers were measured including lycopene, alpha- & beta-carotene, alpha- & gamma-tocopherol, C-reactive protein, triglycerides, total, LDL, and HDL cholesterol.

For the first subsample (BA23) consisting of 2098 samples, DNA methylation levels were measured using the Illumina Infinium HumanMethylation450 BeadChip at the HudsonAlpha Institute of Biotechnology. This platform uses bisulfite conversion to quantify methyltion levels at 485,577 specific CpG sites genome-wide. Samples were prepared according to the standard Illumina protocol, and β methylation values were calculated from the intensity ratio between methylated and total (methylated and unmethylated) probe fluorescence intensities. Methylation data was processed as described in [8]. In order to test the quality of these array measurements, we perform correlation measures with duplicates within this dataset and with a "gold" standard which is an average of many samples previously collected. Correlation between duplicates and with the gold standard were high (r>0.9), indicative of high quality measurements.

The second WHI data set is described in the following.

WHI-EMPC Description

The Women's Health Initiative – Epigenetic Mechanisms of PM-Mediated CVD (WHI-EMPC, AS315) is an ancillary study of epigenetic mechanisms underlying associations between ambient particulate matter (PM) air pollution and cardiovascular disease (CVD) in the Women's Health Initiative clinical trials (CT) cohort. The WHI-EMPC study population is a stratified, random sample of 2,200 WHI CT participants who were examined between 1993 and 2001; had available buffy coat, core analytes, electrocardiograms, and ambient concentrations of PM; but were not taking anti-arrhythmic medications at the time. As such, WHI-EMPC is representative of the larger, multiethnic WHI CT population from which it was sampled: n = 68,132 participants aged 50-79 years who were randomized to hormone therapy, calcium/vitamin D supplementation, and / or dietary modification in 40 U.S. clinical centers at the baseline exam (1993-1998) and re-examined in the fasting state one, three, six, and nine years later [11]. Illumina Infinium HumanMethylation450 BeadChip data from the Northwestern University Genomics Core Facility for WHI-EMPC participants sampled in stages 1a (800 participants), 1b (1200 participants), and 2 (200 participants x 2 samples each) was quality controlled and batch adjusted. Batch adjustment involved applying empirical Bayes methods of adjusting for stage and plate as implemented in ComBat [12].

Lifestyle factors and dietary assessment in the Women's Health Initiative (WHI)

Participants were selected from the WHI, a national study that began in 1993 and enrolled postmenopausal women between the ages of 50-79 years into either randomized clinical trials (RCTs) or into an observational study [13]. Participants completed self-administered questionnaires at baseline which provided personal information on a wide range of topics, including sociodemographic information (age, education, race, income), and current health behaviors (recreational physical activity, tobacco and alcohol exposure, and diet). Participants also visited clinics at baseline where certified Clinical Center staff collected blood specimens and performed anthropometric measurements including weight, height, hip and waist circumferences, and systolic and diastolic blood pressures; body mass index and waist to hip ratio were calculated from these measurements (Supplementary Table 3).

Dietary intake levels were assessed at baseline using the WHI Food Frequency Questionnaire [14]. Briefly, participants were asked to report on dietary habits in the past three months, including intake, frequency, and portion sizes of foods or food groups, along with questions concerning topics such as food preparation practices and types of added fats. Nutrient intake levels were then estimated from these responses. For current drinker, we use the threshold of more than one serving equivalent (14g) within the last 28 days.

Jackson Heart Study

The JHS is a large, population-based observational study evaluating the etiology of cardiovascular, renal, and respiratory diseases among African Americans residing in the three counties (Hinds, Madison, and Rankin) that make up the Jackson, Mississippi metropolitan area [15]. Data and biologic materials have been collected from 5306 participants, including a nested family cohort of 1,498 members of 264 families. The age at enrollment for the unrelated cohort was 35-84 years; the family cohort included related individuals >21 years old. Participants provided extensive medical and social history, had an array of physical and biochemical measurements and diagnostic procedures, and provided genomic DNA during a baseline examination (2000-2004) and two follow-up examinations (2005-2008 and 2009-2012). The study population is characterized by a high prevalence of diabetes, hypertension, obesity, and related disorders. Annual follow-up interviews and cohort surveillance are ongoing.

In our analysis, we used Illumina EPIC array data from n=1756 African Americans (n=1203 women and n=653 men) that were generated as part of project JHS ancillary study ASN0104. The blood samples were collected at the baseline of the study (visit 1). At the time of the blood draw, the individuals ranged from 22 to 93 (median age 57). At the time of the last follow up, 282 individuals were known to be deceased. The median number of years of follow up (time to death or last follow up) was 12.2 years (ranging from 0.14 to 14.5 years).

Framingham Heart Study Offspring Cohort (FHS)

The Framingham Heart Study (FHS) Offspring Cohort began enrollment in 1971 and included 5,124 offspring and spouses of the offspring of the FHS original cohort. Participants were eligible for the current study if they attended the eighth examination cycle (2005-2008) and consented to having their DNA to be used for genetic research. All participants provided written informed consent at the time of each examination visit. The study protocol was approved by the Institutional Review Board at Boston University Medical Center (Boston, MA). The FHS data are available in dbGaP (accession number "phs000724.v2.p9").

Statistical Methods

As for the multi-tissue DNAm age estimator (Horvath 2013) [8], the dependent variable, chronological age, was transformed before carrying out an elastic net regression analysis. Toward this end, the following function F for transforming age was used:

- F(age)=log(age+1)-log(adult.age+1) if age<=adult.age.
- F(age)=(age-adult.age)/(adult.age+1) if age>adult.age.

The parameter "adult.age" was set to 20. Note that F satisfies the following desirable properties: it

- i) is a continuous, monotonically increasing function (which can be inverted),
- ii) has a logarithmic dependence on age until adulthood (here set at 20 years),
- iii) has a linear dependence on age after adulthood (here set to 20),
- iv) is defined for negative ages (i.e. prenatal samples) by adding 1 (year) to age in the logarithm,
- v) it has a continuous first derivative (slope function). In particular the slope at age=adult.age is given by 1/(adult.age+1).

An elastic net regression model (implemented in the glmnet R function) was used to regress a transformed version of age on the beta values in the training data. The glmnet function requires the user to specify two parameters (alpha and beta). Since I used an elastic net predictor, alpha was set to 0.5. But the lambda value of was chosen by applying a 10 fold cross validation to the training data (via the R function cv.glmnet). The elastic net regression results in a linear regression model whose coefficients b_0, b_1, . . . , b_{391} relate to transformed age as follows:

$$F(\text{chronological age})=b_0+b_1CpG_1+\ldots +b_{391}CpG_{391}+\text{error}$$

The coefficient values can be found in Supplementary Dataset 2. Based, on the coefficient values from the regression model, DNAmAge is estimated as follows $DNAm\text{Age}=\text{inverse.}F(b_0+b_1CpG_1+\ldots +b_{391}CpG_{391})$ where inverse.F(.) denotes the mathematical inverse of the function F(.) and is specified as follows.

- anti.F(x)= (1+adult.age)*exp(x)-1 if x<0
- anti.F(x)= (1+adult.age)*x+adult.age if x>=0
- and the parameter adult.age was chosen to be 20.

Thus, the regression model can be used to predict to transformed age value by simply plugging the beta values of the selected CpGs into the formula.

R software code

Assume that dat0 is a data frame of beta values whose first columns contains the cg numbers (probe identifiers) and whose columns contain the beta values.

We recommend that you generate the beta values using a software tool that avoids missing values. For example, the R function "preprocessNoob" in the minfi R package or "preprocessQuantile" in the minfi package.

```
#R functions for transforming age

adult.age1=20

trafo= function(x,adult.age=adult.age1)
{x=(x+1)/(1+adult.age); y=ifelse(x<=1,
log( x),x-1);y }

anti.trafo=
function(x,adult.age=adult.age1) {
ifelse(x<0, (1+adult.age)*exp(x)-1,
(1+adult.age)*x+adult.age) }

datClock=read.csv("path/datSkinClock.cs
v")

selectCpGsClock=is.element(dat0[,1],
as.character(datClock[-1,1]))

datMethClock0=data.frame(t(dat0[selectC
pGsClock ,-1] ))

colnames(datMethClock0)=
as.character(dat0[selectCpGsClock ,1] )

# Reality check: the following output
should only contain numeric values.

# Further, the column names should be
CpG identifiers (cg numbers).

datMethClock0[1:5,1:5]

datMethClock= data.frame(datMethClock0[
as.character(datClock[-1,1])])

# The number of rows should equal the
number of samples (Illumina arrays)

dim(datMethClock)

#Output DNAm age estimator for the skin
& blood clock

DNAmAgeSkinClock=as.numeric(anti.trafo(
datClock$Coef[1]+as.matrix(datMethClock
)%*% as.numeric(datClock$Coef[-1])))
```

REFERENCES

1. Moulson CL, Fong LG, Gardner JM, Farber EA, Go G, Passariello A, Grange DK, Young SG and Miner JH. Increased progerin expression associated with unusual LMNA mutations causes severe progeroid syndromes. Hum Mutat. 2007; 28:882-889.
2. Bar DZ, Arlt MF, Brazier JF, Norris WE, Campbell SE, Chines P, Larrieu D, Jackson SP, Collins FS, Glover TW and Gordon LB. A novel somatic mutation achieves partial rescue in a child with Hutchinson-Gilford progeria syndrome. J Med Genet. 2017; 54:212-216.
3. Bai S, Lozada A, Jones MC, Dietz HC, Dempsey M and Das S. Mandibuloacral Dysplasia Caused by LMNA Mutations and Uniparental Disomy. Case Rep Genet. 2014; 2014:508231.
4. Ahmad Z, Zackai E, Medne L and Garg A. Early onset mandibuloacral dysplasia due to compound heterozygous mutations in ZMPSTE24. Am J Med Genet A. 2010; 152A:2703-2710.
5. Huh CJ, Zhang B, Victor MB, Dahiya S, Batista LF, Horvath S and Yoo AS. Maintenance of age in human neurons generated by microRNA-based neuronal conversion of fibroblasts. Elife. 2016; 5:e18648.
6. Teschendorff AE, Marabita F, Lechner M, Bartlett T, Tegner J, Gomez-Cabrero D and Beck S. A beta-mixture quantile normalization method for correcting probe design bias in Illumina Infinium 450 k DNA methylation data. Bioinformatics. 2013; 29:189-196.
7. Houseman E, Accomando W, Koestler D, Christensen B, Marsit C, Nelson H, Wiencke J and Kelsey K. DNA methylation arrays as surrogate measures of cell mixture distribution. BMC Bioinformatics. 2012; 13:86.
8. Horvath S. DNA methylation age of human tissues and cell types. Genome Biol. 2013; 14:R115.
9. Horvath S and Levine AJ. HIV-1 Infection Accelerates Age According to the Epigenetic Clock. J Infect Dis. 2015; 212:1563-1573.
10. Horvath S, Gurven M, Levine ME, Trumble BC, Kaplan H, Allayee H, Ritz BR, Chen B, Lu AT, Rickabaugh TM, Jamieson BD, Sun D, Li S, et al. An epigenetic clock analysis of race/ethnicity, sex, and coronary heart disease. Genome Biol. 2016; 17:171.
11. Anonymous A. Design of the Women's Health Initiative clinical trial and observational study. The Women's Health Initiative Study Group. Control Clin Trials. 1998; 1961-109.
12. Johnson WE, Rabinovic A and Li C. Adjusting batch effects in microarray expression data using Empirical Bayes methods. Biostatistics. 2007; 8:118-127.
13. WHI. Design of the Women's Health Initiative clinical trial and observational study. Controlled clinical trials. 1998; 19:61-109.
14. Patterson RE, Kristal AR, Tinker LF, Carter RA, Bolton MP and Agurs-Collins T. Measurement characteristics of the Women's Health Initiative food frequency questionnaire. Annals of epidemiology. 1999; 9:178-187.
15. Taylor Jr HA, Wilson JG, Jones DW, Sarpong DF, Srinivasan A, Garrison RJ, Nelson C and Wyatt SB. Toward resolution of cardiovascular health disparities in African Americans: design and methods of the Jackson Heart Study. Ethn Dis. 2005; 15(4 Suppl 6):S6-4.
16. Hannum G, Guinney J, Zhao L, Zhang L, Hughes G, Sadda S, Klotzle B, Bibikova M, Fan JB, Gao Y, Deconde R, Chen M, Rajapakse I, et al. Genome-wide methylation profiles reveal quantitative views of human aging rates. Mol Cell. 2013; 49:359-367.
17. Chen BH, Marioni RE, Colicino E, Peters MJ, Ward-Caviness CK, Tsai PC, Roetker NS, Just AC, Demerath EW, Guan W, Bressler J, Fornage M, Studenski S, et al. DNA methylation-based measures of biological age: meta-analysis predicting time to death. Aging (Albany NY). 2016; 8:1844-1865.
18. Horvath S and Ritz BR. Increased epigenetic age and granulocyte counts in the blood of Parkinson's disease patients. Aging (Albany NY). 2015; 7:1130-1142.
19. Rojas D, Rager JE, Smeester L, Bailey KA, Drobna Z, Rubio-Andrade M, Styblo M, Garcia-Vargas G and Fry RC. Prenatal arsenic exposure and the epigenome: identifying sites of 5-methylcytosine alterations that predict functional changes in gene expression in newborn cord blood and subsequent birth outcomes. Toxicol Sci. 2015; 143:97-106.
20. Knight AK, Craig JM, Theda C, Bækvad-Hansen M, Bybjerg-Grauholm J, Hansen CS, Hollegaard MV, Hougaard DM, Mortensen PB, Weinsheimer SM, Werge TM, Brennan PA, Cubells JF, et al. An epigenetic clock for gestational age at birth based on blood methylation data. Genome Biology. 2016; 17:206.
21. Kozlenkov A, Jaffe AE, Timashpolsky A, Apontes P, Rudchenko S, Barbu M, Byne W, Hurd YL, Horvath S and Dracheva S. DNA Methylation Profiling of Human Prefrontal Cortex Neurons in Heroin Users Shows Significant Difference between Genomic Contexts of Hyper- and Hypomethylation and a Younger Epigenetic Age. Genes (Basel). 2017; 8.

22. Horvath S, Mah V, Lu AT, Woo JS, Choi OW, Jasinska AJ, Riancho JA, Tung S, Coles NS, Braun J, Vinters HV and Coles LS. The cerebellum ages slowly according to the epigenetic clock. Aging (Albany NY). 2015; 7:294-306.

23. Ahrens M, Ammerpohl O, von Schönfels W, Kolarova J, Bens S, Itzel T, Teufel A, Herrmann A, Brosch M, Hinrichsen H, Erhart W, Egberts J, Sipos B, et al. DNA Methylation Analysis in Nonalcoholic Fatty Liver Disease Suggests Distinct Disease-Specific and Remodeling Signatures after Bariatric Surgery. Cell Metabolism. 2013; 18:296-302.

24. Horvath S, Erhart W, Brosch M, Ammerpohl O, von Schonfels W, Ahrens M, Heits N, Bell JT, Tsai PC, Spector TD, Deloukas P, Siebert R, Sipos B, et al. Obesity accelerates epigenetic aging of human liver. Proc Natl Acad Sci U S A. 2014; 111:15538-15543.

25. Viechtbauer W. Conducting Meta-Analyses in R with the metafor Package. J Statistical Software. 2010; 36:1-48.

26. Bacalini MG, Gentilini D, Boattini A, Giampieri E, Pirazzini C, Giuliani C, Fontanesi E, Scurti M, Remondini D, Capri M, Cocchi G, Ghezzo A, Del Rio A, et al. Identification of a DNA methylation signature in blood cells from persons with Down Syndrome. Aging (Albany NY). 2015; 7:82-96.

27. Horvath S, Garagnani P, Bacalini MG, Pirazzini C, Salvioli S, Gentilini D, Di Blasio AM, Giuliani C, Tung S, Vinters HV and Franceschi C. Accelerated epigenetic aging in Down syndrome. Aging Cell. 2015; 14:491-495.

28. Quach A, Levine ME, Tanaka T, Lu AT, Chen BH, Ferrucci L, Ritz B, Bandinelli S, Neuhouser ML, Beasley JM, Snetselaar L, Wallace RB, Tsao PS, et al. Epigenetic clock analysis of diet, exercise, education, and lifestyle factors. Aging (Albany NY). 2017; 9:419-446.
