# Supplementary material for: Epigenetic clock for skin and blood cells applied to Hutchinson Gilford Progeria Syndrome and ex vivo studies
Source: Aging (Albany NY). 2018 Jul 26;10(7):1758–75. doi: 10.18632/aging.101508 (PMC6075434; doi:10.18632/aging.101508)
Supplement: Supplementary Figures [file aging-10-101508-s002.pdf]

## SUPPLEMENTARY FIGURES

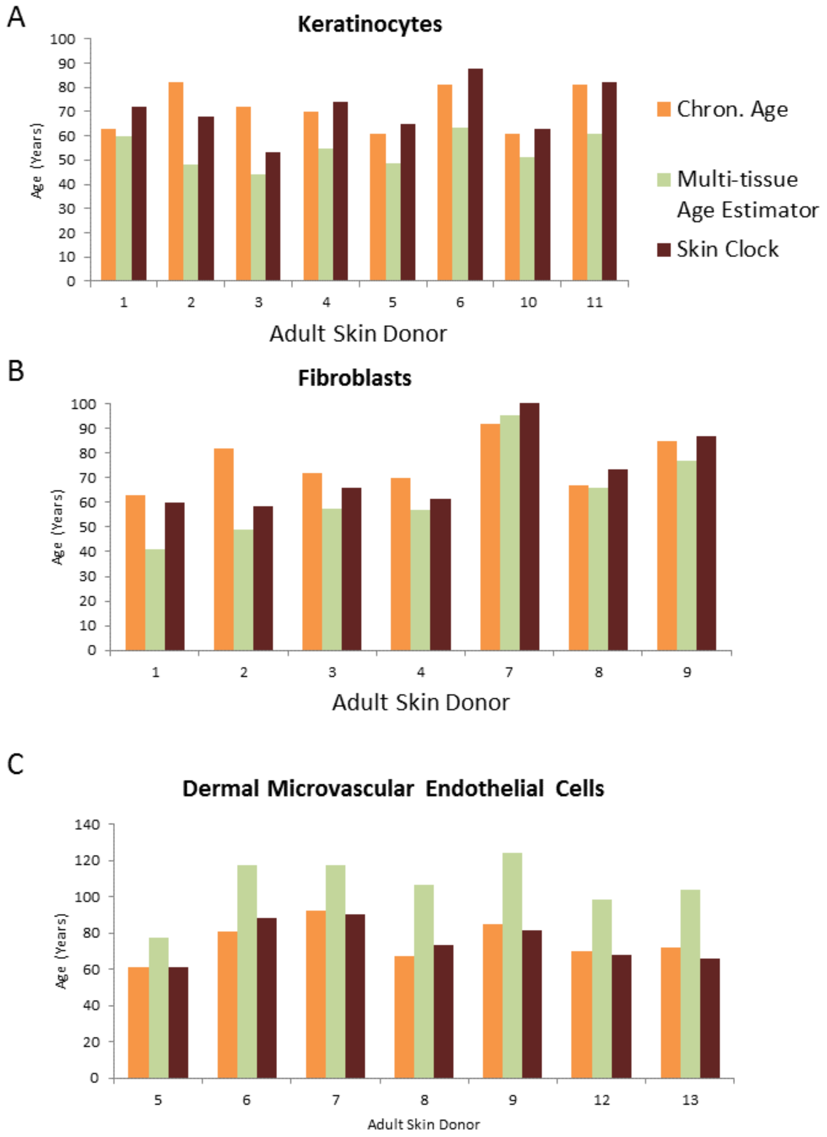


**Supplementary Figure S1. Comparing the new skin & blood clock with the pan-tissue age estimator in different cell types.** The y-axis reports chronological age estimates based on DNA methylation levels from (**A**) keratinocytes, (**B**) fibroblasts and (**C**) microvascular endothelial cells. The x-axis corresponds to different donors whose chronological ages are indicated by the orange bars. The age estimates of the skin & blood clock and the pan-tissue clock are colored in brown and green, respectively.

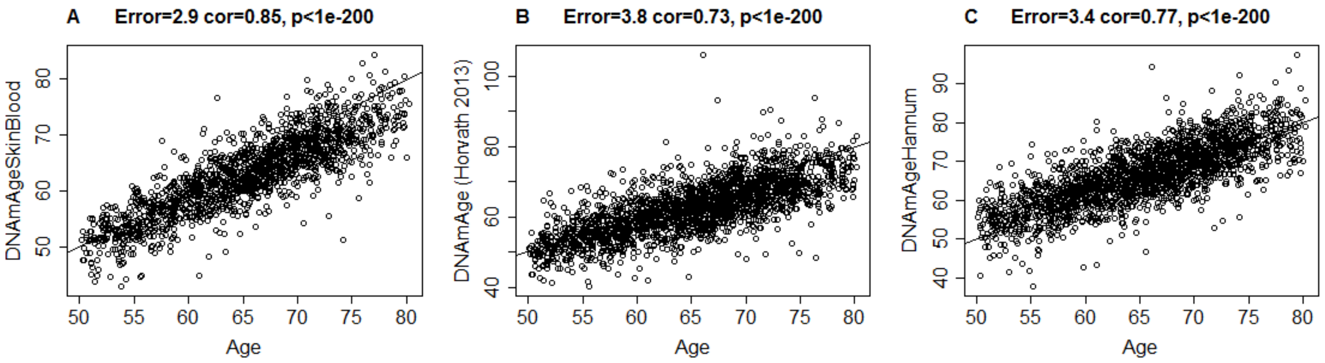


**Supplementary Figure S2. Accuracy of different DNAm age estimators in blood from the WHI**. Age at blood draw (x-axis) versus DNAm age estimates from (**A**) the novel skin & blood clock, (**B**) the pan-tissue DNAm age estimator (Horvath 2013) [8], (**C**) DNAm age estimator by Hannum (2013)[16]. The DNA methylation data from participants of the Women's Health Initiative are described in [10, 17]. The error is defined as the median absolute deviation between chronological age and the age estimate.

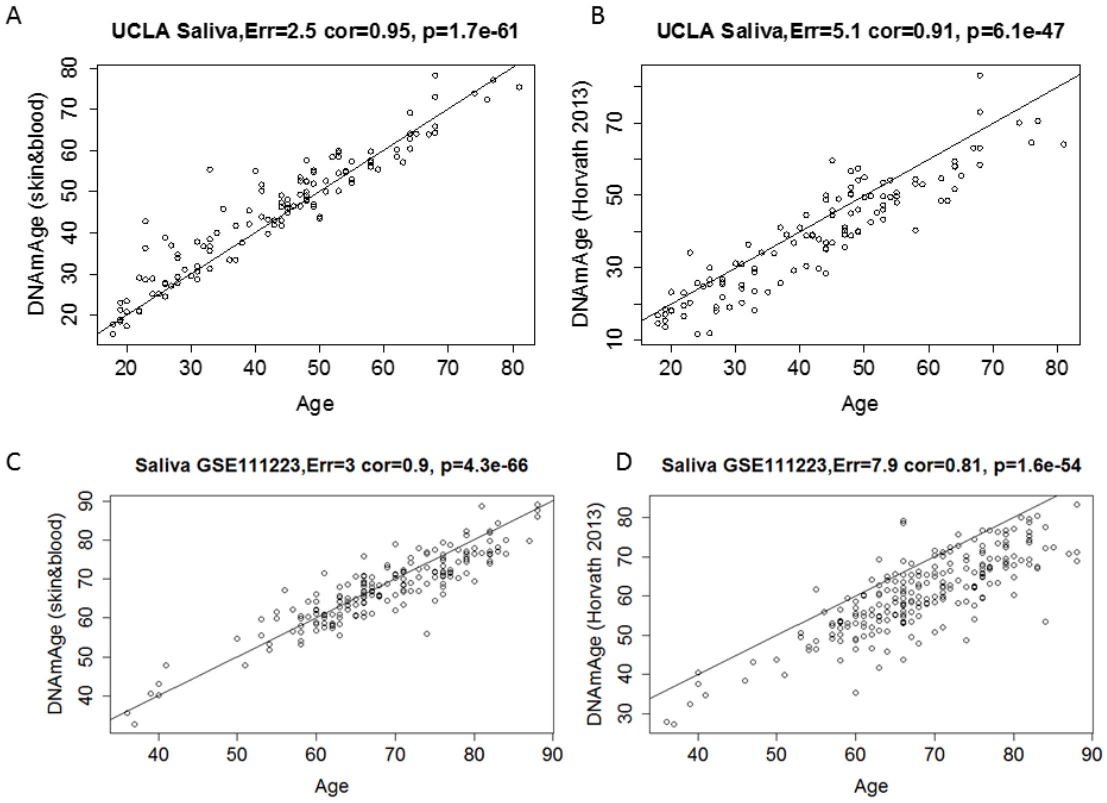


**Supplementary Figure S3. Accuracy of different DNAm age estimators in two different saliva data sets.** Age at the collection of saliva samples (via a spit cup) (x-axis) versus DNAm age estimates from (**A**,**C**) the novel skin & blood clock (**B**,**D**), the pan-tissue DNAm age estimator (Horvath 2013) [8]. The error is defined as the median absolute deviation between chronological age and the age estimate. Panels on the first and second row correspond to (**A**,**B**) an Illumina 450K DNA methylation data set from UCLA and (**C**,**D**) a publicly available DNA methylation data set (Gene Expression Omnibus identifier GSE111223) described in Horvath and Ritz 2015 [18].

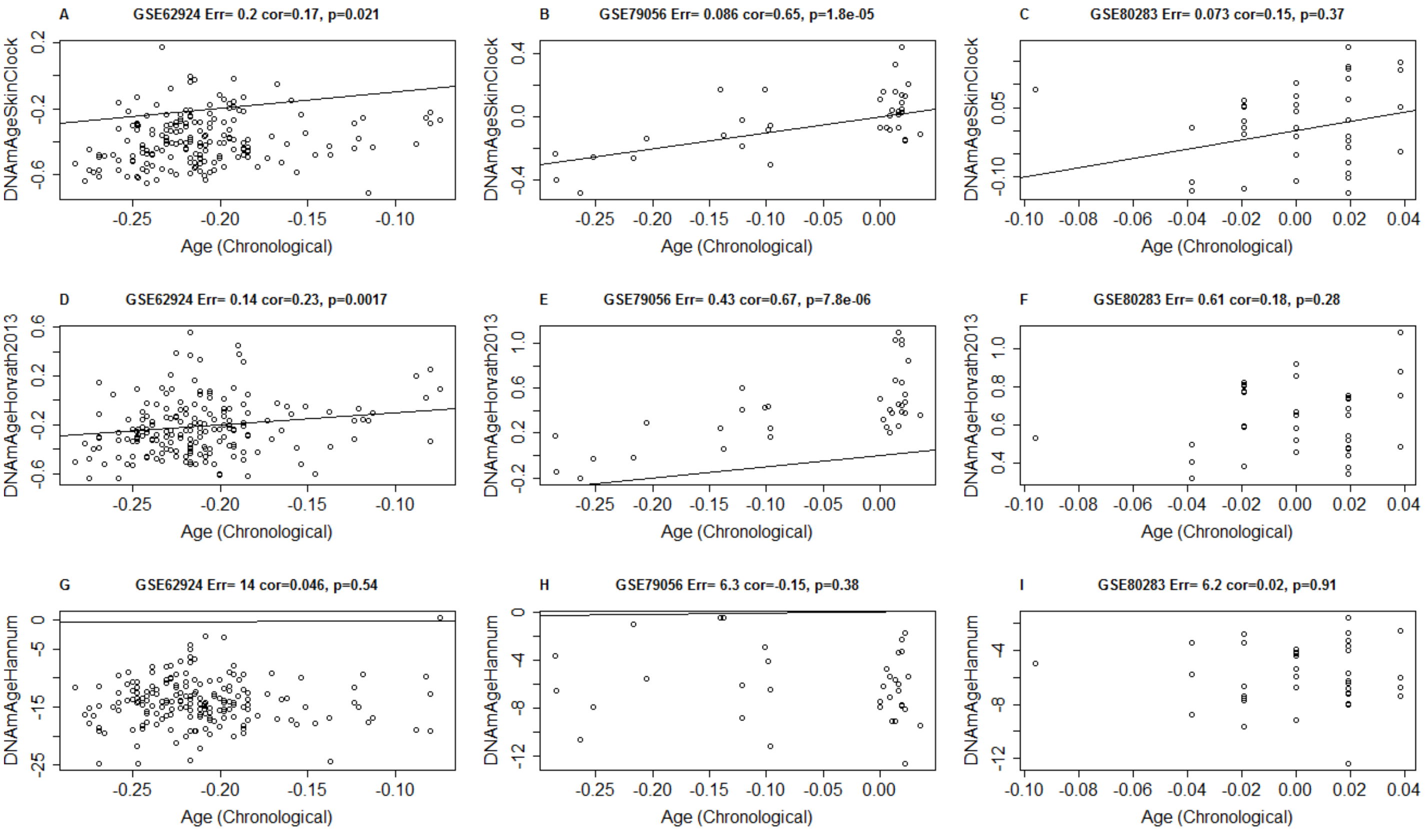


**Supplementary Figure S4. Gestational age versus different DNAm age estimates from blood.** Age blood draw in units of years (x-axis) versus DNAm age estimates from (**A**,**B**,**C**) the novel skin & blood clock (**D**,**E**,**F**), the pan-tissue DNAm age estimator (Horvath 2013) [8], and (**G**,**H**,**I**) the Hannum (2013) [16] clock. Gestational Week was translated into units of years using the following formula Age=(Gestational Week-39)/52. The error is defined as the median absolute deviation between chronological age and the age estimate. Panels in the different columns correspond to three publicly available data sets: (**A**,**D**,**G**) GEO identifier GSE62924 [19], (**B**,**E**,**H**) Nashville birth cohort (GSE79056 [20]), (**C**,**F**,**I**) Victorian Infant Collaborative Study GSE80283 (contributed by Doyle L, Chong J, Craig J).

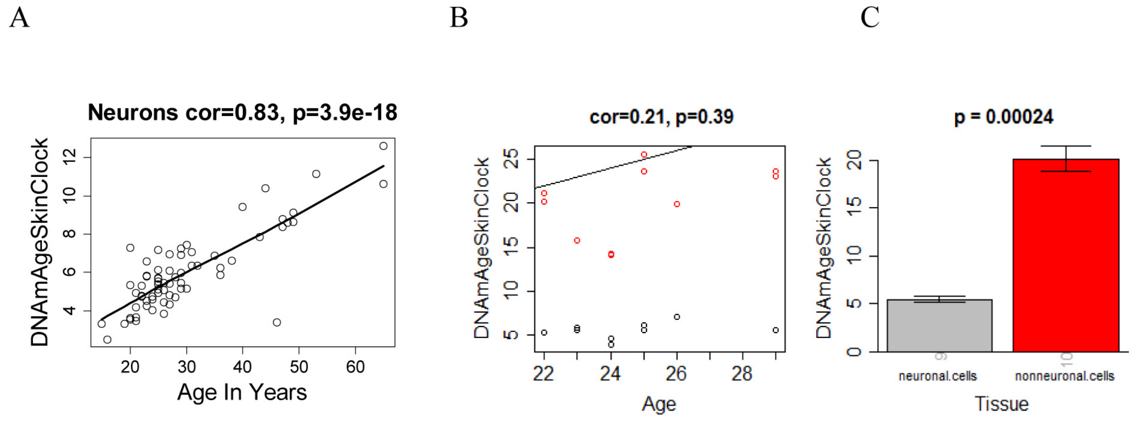


**Supplementary Figure S5. Skin & blood clock analysis sorted neurons and glia.** The DNA methylation data are described in [21]. Left panel: we used n=87 neuronal samples from individuals (aged between 15 and 65 years, mean age=31). The neuronal data were generated using Nuclei Separation by Fluorescence Activated Nuclei Sorting (FANS) from frozen autopsy brain samples. The nuclei of different cell types remain intact in frozen autopsy brain samples. Antibodies against the RNA-binding protein NeuN, which is expressed exclusively in the neuronal nuclei, have been used to separate neuronal from glial nuclei using FANS [21]. Middle panel: DNAm age (y-axis) versus chronological age for neurons (colored black) and glia cells (colored red) from the same individuals. Note that the red dots (glia) lie above the black dots, which indicates that glial cells are epigenetically older than neurons. Right panel: mean DNAm age estimate versus cell type.

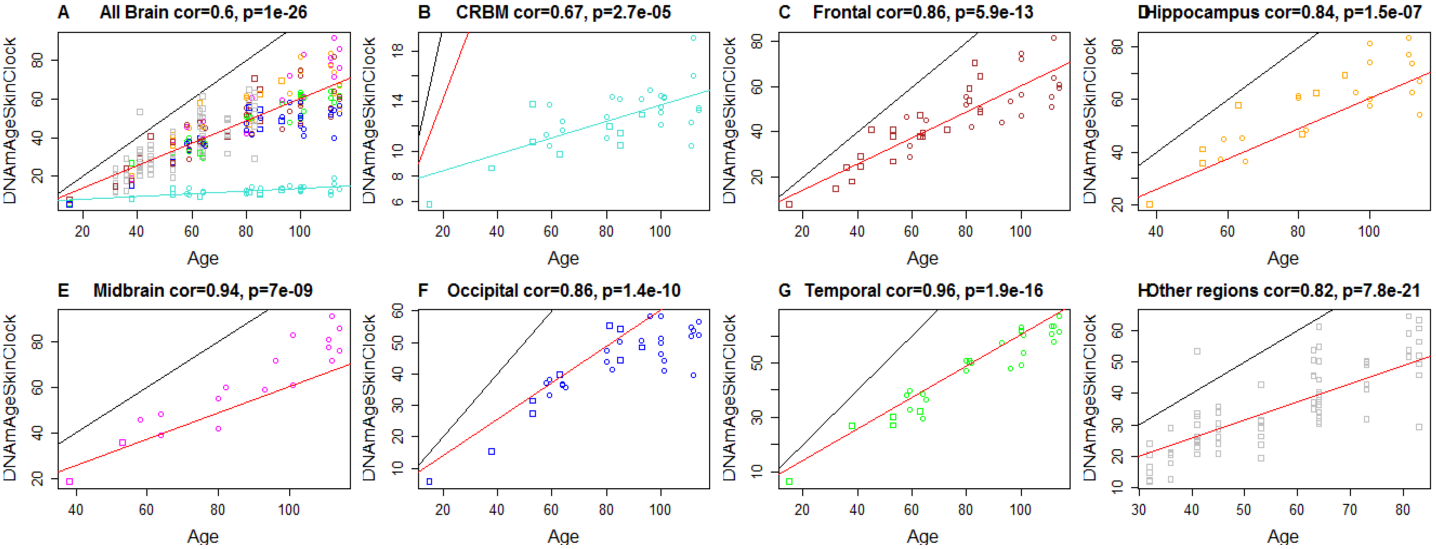


**Supplementary Figure S6. Skin & blood clock analysis of different brain regions**. The Illumina 450L data from different brain regions are described in [22]. Briefly, n=260 arrays were generated from 39 individuals (19 females). We profiled the following brain regions: caudate nucleus (n = 12 arrays), cingulate gyrus (n=12 arrays), cerebellum (32), hippocampus (25), inferior parietal cortex (11), left frontal lobe (9), left occipital cortex (12), left temporal cortex (18), midbrain (18), middle frontal gyrus (12), motor cortex (12), right frontal lobe (20), right occipital cortex (21), right temporal cortex (11), sensory cortex (12), superior parietal cortex (12), and visual cortex (11). Twenty-one individuals presented with Alzheimer's Disease (AD) whereas 18 individuals did not have any neurodegenerative disease. We ignored AD status because no significant epigenetic age acceleration effect associated with AD could be detected. The red line is a regression line. The black line depicts the diagonal line y=x.

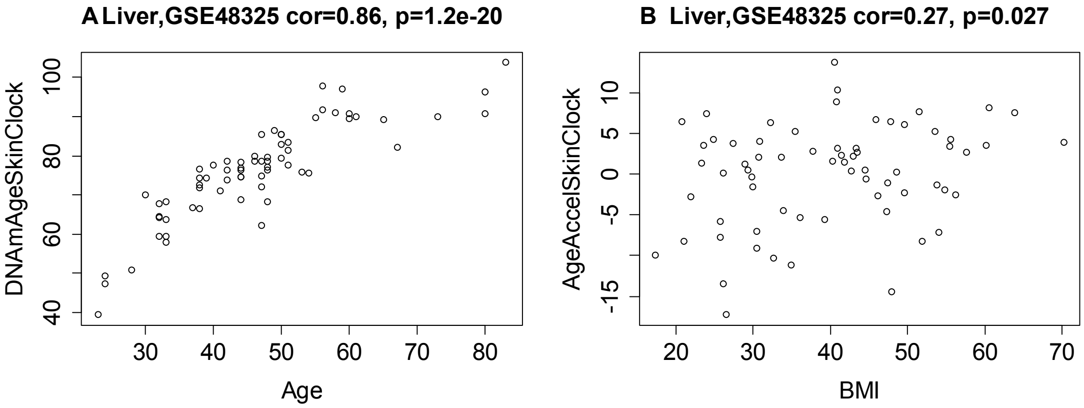


**Supplementary Figure S7. Skin & blood clock analysis of liver tissue samples from obese individuals**. (**A**) According to the skin & blood clock, DNAm age of liver tissue is highly correlated with chronological age (x-axis). (**B**) The corresponding measure of epigenetic age acceleration correlates with body mass index (x-axis). The publicly available liver methylation data (GSE48325) are described in [23, 24].

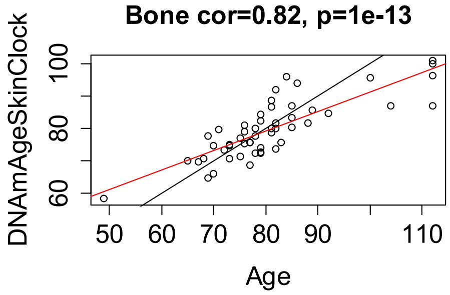


**Supplementary Figure S8. Skin & blood clock analysis of bone samples.** The bone data are described in [18]. The trabecular bone pieces were obtained from the central part of the femoral head of Spanish (Caucasian) patients with hip fractures (due to osteoporosis) or individuals with osteoarthritis. Osteoarthritis status was not related to DNA methylation age in this data set. We also included the bone samples from the supercentenarian (age 112) described elsewhere.

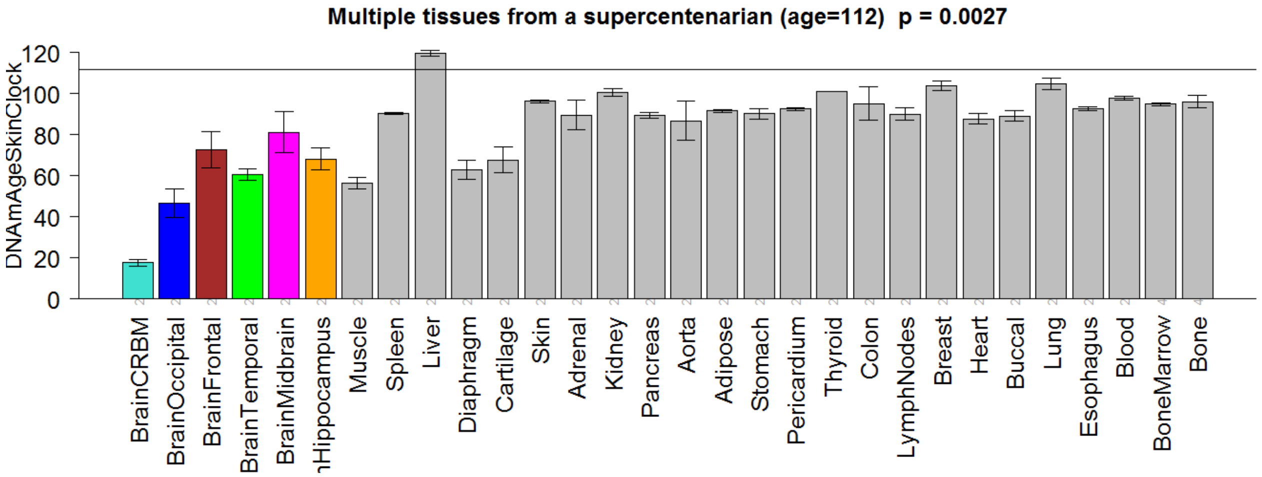


**Supplementary Figure S9. Skin & blood clock analysis of 30 different body parts of a 112 year old woman.** DNAm age of postmortem tissue samples (y-axis) versus body part (x-axis). These data are described in [22]. For each body part, we considered at least 2 replicate samples. The black horizontal line corresponds to the actual chronological age of 112 years. Note that the cerebellum ages more slowly than other body parts which echoes the findings in [22].

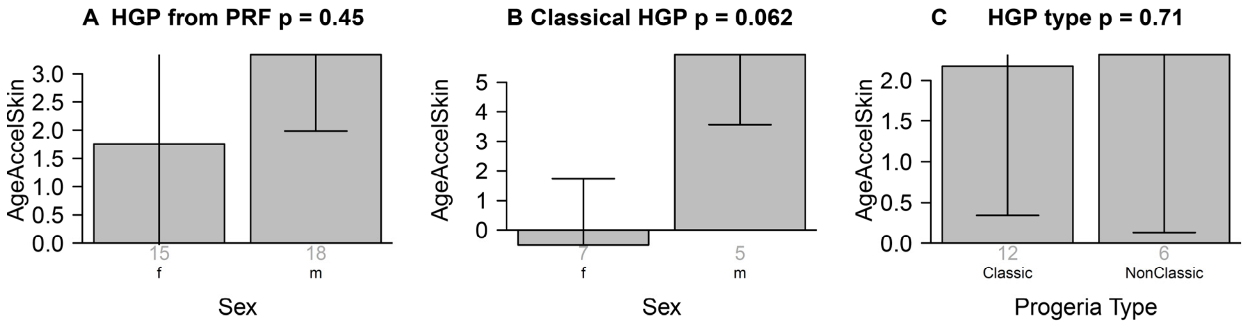


**Supplementary Figure S10. Detailed analysis of HGPS fibroblast samples from the Progeria Research Foundation (PRF).** (**A**) Sex (x-axis) versus epigenetic age acceleration in all HGP samples from the PRF (Table 2). (**B**) Sex versus epigenetic age acceleration in classical HGP samples. (**C**) Epigenetic age acceleration does not relate to progeria type (classical versus non-classical). Each bar plot reports the findings from a non-parametric group comparison test (Kruskal Wallis test). Each bar plot depicts the mean value of age acceleration and one standard error (error bars).

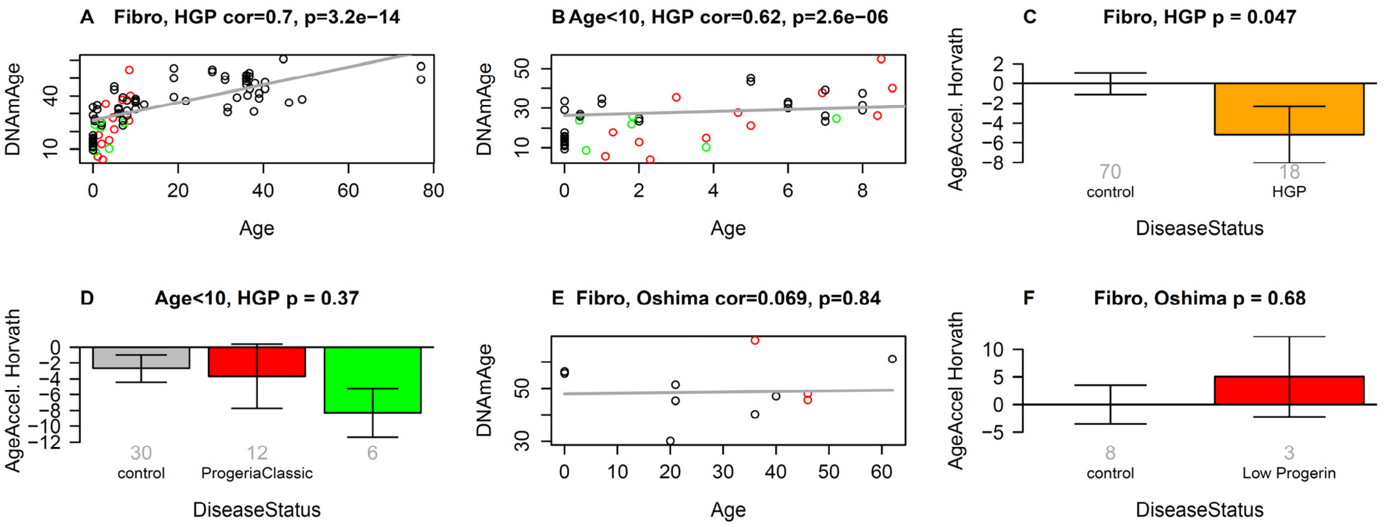


**Supplementary Figure S11. Pan-tissue clock analysis of fibroblasts from HGP individuals of the Progeria Research Foundation**. (**A**,**B**) The pan-tissue clock (Horvath 2013) [8] was used to estimate DNAm age (y-axis) in fibroblasts from HGP individuals and controls. (**A**) All individuals. (**B**) Children younger than 10 years old. Dots are colored by disease status: red=classical progeria, green=non-classical progeria, black=controls. The grey line corresponds to a regression line through control individuals. The epigenetic age acceleration effect for each individual (point) corresponds to the vertical distance to the black regression line. The fact that red and green points tend to lie below the grey line indicates that HGP cases exhibit suggestive accelerated epigenetic aging effect. (**C**) Mean epigenetic age acceleration (y-axis) versus HGP status. By definition, the mean age negative acceleration measure in controls is zero. The title of the bar plots also reports a P-value from a nonparametric group comparison test (Kruskal Wallis test). Each bar plot reports 1 standard error. (**D**) Epigenetic age acceleration (y-axis) versus disease status in individuals younger than 10. (**E**, **F**) report results for fibroblast samples from atypical Werner syndrome cases (low progerin) provided by co-author Junko Oshima. (**E**) DNAm age versus chronological age for atypical Werner syndrome samples (colored in red) and controls (colored in black). (**F**) Epigenetic age acceleration versus disease status.

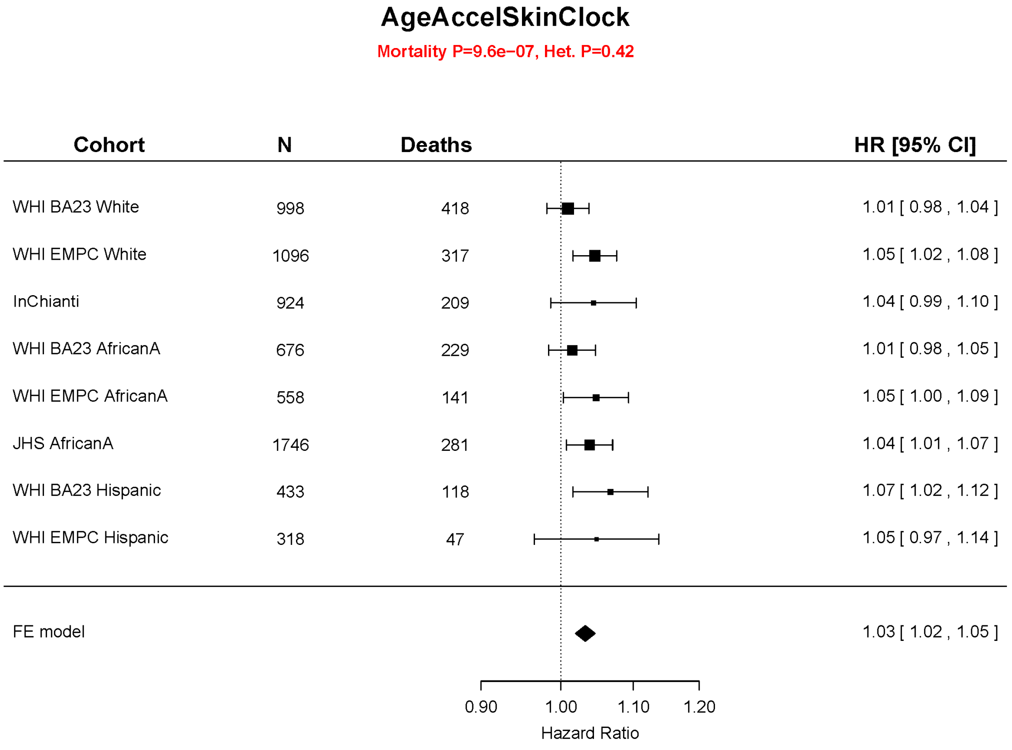


**Supplementary Figure S12. Univariate Cox regression meta-analysis of all-cause mortality (time to death).** A univariate Cox regression model was used to relate the censored survival time (time to all-cause mortality) to epigenetic age acceleration (according to the skin & blood clock). The rows correspond to the different cohorts/racial groups. Each row depicts the hazard ratio and a 95% confidence interval. The coefficient estimates from the respective studies were meta-analyzed using a fixed-effect model weighted by inverse variance (implemented in the "metafor" R package [25]. The meta analysis p value (red sub-title) is highly significant p=9.6E-7. The p-value of the heterogeneity test (Cochran's Q-test) is not significant because the cohort-specific estimates do not differ substantially (which is a desirable finding).

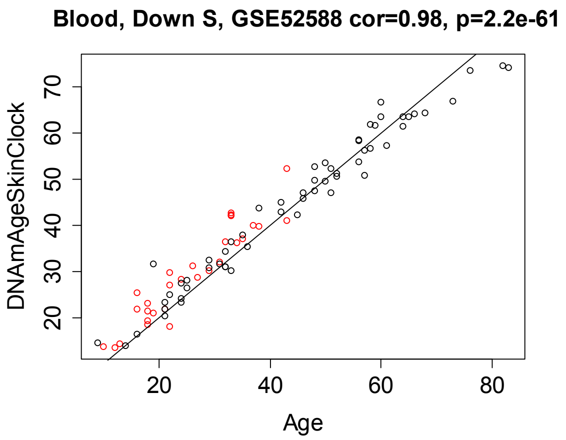


**Supplementary Figure S13. Skin & blood clock analysis of blood samples from individuals with Down syndrome (red dots) and controls (black dots).** The scatter plot relates the DNAm age estimate of each sample (y-axis) to chronological age (x-axis). The black line corresponds to y=x. The data are publicly available from Gene Expression Omnibus (GSE52588) and are described in [26, 27]. The data involved blood samples from 29 individuals with DS, their mothers and their unaffected siblings. This design allows adjustment for possible confounding effects on DNA methylation patterns deriving from genetic and environmental (lifestyle) factors within families. To properly account for the family relationships, we also fit a mixed effects model to the 29 discordant sib pairs. Specifically, DNAm age was regressed on DS status, chronological age, and a random effects term (intercept) that encoded the sib ship. Using this model, we found that DS status significantly affected DNAm age (P = 0.034), raising it by 1.84 years (standard error  0.877).

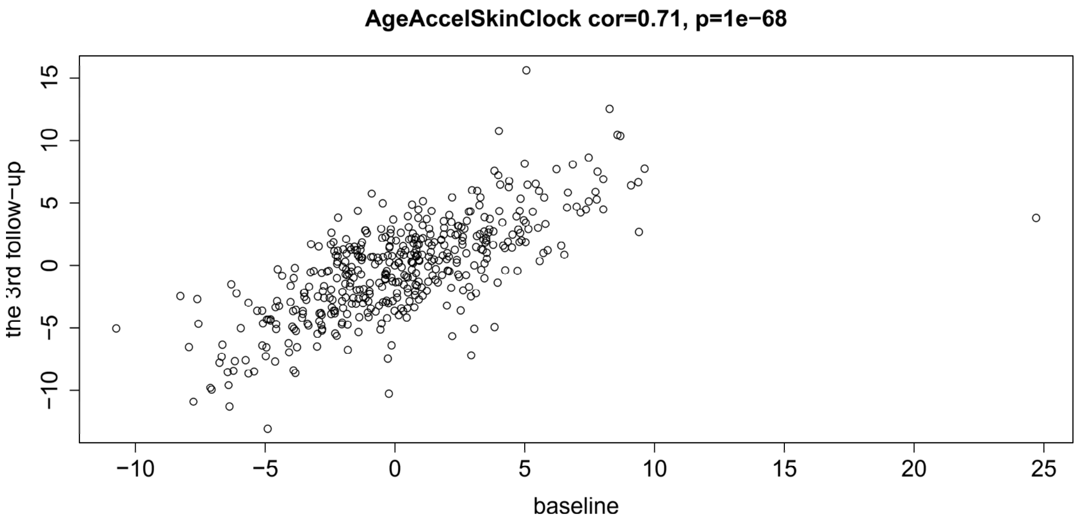


**Supplementary Figure S14. Longitudinal follow up study in the InCHIANTI cohort**. AgeAcceleration at baseline (x-axis) and nine years later (y-axis).
