# Supplementary material for: Epigenetic clock for skin and blood cells applied to Hutchinson Gilford Progeria Syndrome and ex vivo studies
Source: Aging (Albany NY). 2018 Jul 26;10(7):1758–75. doi: 10.18632/aging.101508 (PMC6075434; doi:10.18632/aging.101508)
Supplement: Supplementary Tables [file aging-10-101508-s003.pdf]

SUPPLEMENTARY TABLES

Supplementary Table 1. Multivariate linear regression analysis of HPG using the pan-tissue DNAm age estimator (Horvath 2013) [8].

Outcome: Pan-tissue DNAmAge (Horvath 2013)						
	Data: All, n=88			Data: Age<10, n=44		
	Coef	SE	P-value	Coef	SE	P-value
**Age**	1.10	1.57E-1	8.85E-10	1.82	2.02	0.37
**Age^2**	-9.98E-3	2.54E-3	1.86E-4	5.37E-2	2.32E-1	0.82
**PopulationDoublingLevel**	-4.75E-1	2.01E-1	2.07E-2	-4.25E-1	3.19E-1	0.192
**HGP.Disease**	-2.83	2.77	3.10E-1	-2.88	3.02	0.35

DNAm age is regressed on chronological age, population doubling levels, and HGP disease status. The table reports estimates of the regression coefficients and corresponding standard errors, Wald test P-values. The left panel and right panel report the results for all n=88 fibroblast samples and for n=44 samples from children (younger than 10 years old), respectively.

Supplementary Table 2. This is an expanded version of Table 2 which also includes the parental controls. The comma delimited Excel file will be uploaded as a separate data file. The link to these data is provided in Full text version (Dataset 1).

Dataset 2 presents Excel file (comma delimited csv file) which reports the CpGs and coefficient values for the skin and blood clock. However, the resulting age estimate needs to be transformed as detailed in our Methods supplement. The corresponding R software code can also be found in the Supplementary Material (word document). The link to the data is provided in Full text version (Dataset 2).

Supplementary Table 3. Cross sectional correlations of various variables (diet, lifestyle, demographic) with epigenetic age acceleration in the WHI. Correlations (bicor, biweight midcorrelation) between select variables epigenetic age acceleration based on the new skin & blood clock. The upper panel ignores racial status. Lower panels report results for women of European ancestry (Caucasian), African Ancestry, and Hispanic ancestry. The self-reported race variable was subsequently verified with genetic ancestry markers (i.e. SNP data). The entries are colored according to their magnitude with positive correlations in red, negative correlations in blue, and statistical significance (p-values) in green. Blood biomarkers were measured from fasting plasma collected at baseline. Food groups and nutrients are inclusive, including all types and all preparation methods, e.g. folic acid includes synthetic and natural, dairy includes cheese and all types of milk. The individual variables (rows) are explained in [28].

In all women (irrespective of race)

			AASkin	
		n	bicor	p
Diet	log2(Total energy)	3700	-0.03	0.04
	Carbohydrate	3700	-0.01	0.67
	Protein	3700	-0.03	0.12
	Fat	3700	0.03	0.08
	log2(1+Red meat)	3700	0.01	0.37
	log2(1+Poultry)	3700	-0.06	3E-4
	log2(1+Fish)	3700	-0.06	2E-4
	log2(1+Dairy)	3700	-0.01	0.41
	log2(1+Whole grains)	3700	-0.02	0.30
	log2(1+Nuts)	3700	0.00	0.87
	log2(Fruits)	3700	-0.04	0.01
	log2(Vegetables)	3700	-0.04	0.01
Dietary biomarkers	Retinol	2267	0.02	0.31
	Mean carotenoids	2266	-0.09	8E-6
	Lycopene	2267	-0.05	0.02
	log2(alpha-Carotene)	2267	-0.05	0.01
	log2(beta-Carotene)	2266	-0.07	4E-4
	log2(Lutein+Zeaxanthin)	2267	-0.06	0.01
	log2(beta-Cryptoxanthin)	2267	-0.11	2E-7
	log2(alpha-Tocopherol)	2267	-0.04	0.08
	log2(gamma-Tocopherol)	2267	0.06	3E-3
Measurements	log2(C-reactive protein)	2809	0.06	1E-3
	log2(Insulin)	4042	0.05	2E-3
	log2(Glucose)	4144	0.04	0.01
	log2(Triglyceride)	4148	0.05	3E-3
	Total cholesterol	4148	0.00	0.97
	LDL cholesterol	4084	0.00	0.87
	HDL cholesterol	4145	-0.05	5E-4
	log2(Creatinine)	2748	0.00	0.93
	Systolic blood pressure	4177	0.05	2E-3
	Diastolic blood pressure	4178	0.02	0.21
	log2(Waist / hip ratio)	4037	0.06	2E-4
Lifestyle	BMI	4145	0.04	0.01
	Education	4143	-0.06	6E-5
	Income	4054	-0.04	0.02
	log2(1+Exercise)	3914	-0.05	4E-3
	Current smoker	2321	0.04	0.06
	log2(1+Alcohol)	3700	-0.02	0.14

In all women (irrespective of race)

			AASkin	
		n	bicor	p
Diet	log2(Total energy)	3700	-0.03	0.04
	Carbohydrate	3700	-0.01	0.67
	Protein	3700	-0.03	0.12
	Fat	3700	0.03	0.08
	log2(1+Red meat)	3700	0.01	0.37
	log2(1+Poultry)	3700	-0.06	3E-4
	log2(1+Fish)	3700	-0.06	2E-4
	log2(1+Dairy)	3700	-0.01	0.41
	log2(1+Whole grains)	3700	-0.02	0.30
	log2(1+Nuts)	3700	0.00	0.87
	log2(Fruits)	3700	-0.04	0.01
	log2(Vegetables)	3700	-0.04	0.01
Dietary biomarkers	Retinol	2267	0.02	0.31
	Mean carotenoids	2266	-0.09	8E-6
	Lycopene	2267	-0.05	0.02
	log2(alpha-Carotene)	2267	-0.05	0.01
	log2(beta-Carotene)	2266	-0.07	4E-4
	log2(Lutein+Zeaxanthin)	2267	-0.06	0.01
	log2(beta-Cryptoxanthin)	2267	-0.11	2E-7
	log2(alpha-Tocopherol)	2267	-0.04	0.08
	log2(gamma-Tocopherol)	2267	0.06	3E-3
Measurements	log2(C-reactive protein)	2809	0.06	1E-3
	log2(Insulin)	4042	0.05	2E-3
	log2(Glucose)	4144	0.04	0.01
	log2(Triglyceride)	4148	0.05	3E-3
	Total cholesterol	4148	0.00	0.97
	LDL cholesterol	4084	0.00	0.87
	HDL cholesterol	4145	-0.05	5E-4
	log2(Creatinine)	2748	0.00	0.93
	Systolic blood pressure	4177	0.05	2E-3
	Diastolic blood pressure	4178	0.02	0.21
	log2(Waist / hip ratio)	4037	0.06	2E-4
Lifestyle	BMI	4145	0.04	0.01
	Education	4143	-0.06	6E-5
	Income	4054	-0.04	0.02
	log2(1+Exercise)	3914	-0.05	4E-3
	Current smoker	2321	0.04	0.06
	log2(1+Alcohol)	3700	-0.02	0.14

In African American Women

African American					
	Diet	log2(Total energy)	1079	0.00	0.89
		Carbohydrate	1079	0.02	0.60
		Protein	1079	-0.04	0.15
		Fat	1079	-0.02	0.61
		log2(1+Red meat)	1079	0.05	0.11
		log2(1+Poultry)	1079	-0.01	0.86
		log2(1+Fish)	1079	-0.07	0.02
		log2(1+Dairy)	1079	-0.01	0.76
		log2(1+Whole grains)	1079	-0.01	0.76
		log2(1+Nuts)	1079	0.01	0.74
		log2(Fruits)	1079	0.01	0.70
		log2(Vegetables)	1079	-0.02	0.46
	Dietary biomarkers	Retinol	596	0.01	0.76
		Mean carotenoids	595	-0.08	0.06
		Lycopene	596	-0.07	0.08
		log2(alpha-Carotene)	596	-0.08	0.04
		log2(beta-Carotene)	595	-0.07	0.11
		log2(Lutein+Zeaxanthin)	596	-0.05	0.23
		log2(beta-Cryptoxanthin)	596	-0.04	0.37
		log2(alpha-Tocopherol)	596	0.02	0.66
		log2(gamma-Tocopherol)	596	0.04	0.37
	Measurements	log2(C-reactive protein)	912	0.05	0.14
		log2(Insulin)	1162	0.06	0.04
		log2(Glucose)	1179	0.05	0.12
		log2(Triglyceride)	1179	0.04	0.15
		Total cholesterol	1179	0.00	0.93
		LDL cholesterol	1171	0.01	0.84
		HDL cholesterol	1179	-0.05	0.06
		log2(Creatinine)	900	0.08	0.02
		Systolic blood pressure	1193	0.09	2E-3
		Diastolic blood pressure	1193	0.01	0.74
		log2(Waist / hip ratio)	1156	0.06	0.03
		BMI	1182	0.05	0.06
	Lifestyle	Education	1180	-0.06	0.03
		Income	1154	-0.03	0.33
		log2(1+Exercise)	1159	-0.08	4E-3
		Current smoker	681	0.00	0.91
		log2(1+Alcohol)	1079	-0.04	0.21

In Hispanic Women

Hispanic					
	Diet	log2(Total energy)	636	-0.09	0.03
		Carbohydrate	636	-0.05	0.25
		Protein	636	0.01	0.82
		Fat	636	0.06	0.16
		log2(1+Red meat)	636	0.00	0.97
		log2(1+Poultry)	636	-0.10	0.01
		log2(1+Fish)	636	-0.10	0.01
		log2(1+Dairy)	636	-0.06	0.16
		log2(1+Whole grains)	636	-0.07	0.08
		log2(1+Nuts)	636	0.01	0.83
		log2(Fruits)	636	-0.07	0.09
		log2(Vegetables)	636	-0.08	0.06
	Dietary biomarkers	Retinol	346	-0.05	0.33
		Mean carotenoids	346	-0.04	0.47
		Lycopene	346	-0.03	0.54
		log2(alpha-Carotene)	346	-0.03	0.59
		log2(beta-Carotene)	346	0.00	0.99
		log2(Lutein+Zeaxanthin)	346	-0.03	0.62
		log2(beta-Cryptoxanthin)	346	-0.03	0.57
		log2(alpha-Tocopherol)	346	-0.03	0.53
		log2(gamma-Tocopherol)	346	0.02	0.71
	Measurements	log2(C-reactive protein)	534	0.11	0.01
		log2(Insulin)	702	0.06	0.12
		log2(Glucose)	715	0.06	0.14
		log2(Triglyceride)	715	0.04	0.30
		Total cholesterol	715	0.00	0.89
		LDL cholesterol	702	-0.01	0.79
		HDL cholesterol	715	-0.06	0.10
		log2(Creatinine)	520	-0.04	0.42
		Systolic blood pressure	719	-0.01	0.89
		Diastolic blood pressure	719	-0.02	0.63
		log2(Waist / hip ratio)	704	0.06	0.09
		BMI	712	0.00	0.94
	Lifestyle	Education	708	-0.01	0.79
		Income	693	-0.05	0.17
		log2(1+Exercise)	690	-0.01	0.74
		Current smoker	344	0.05	0.33
		log2(1+Alcohol)	636	0.00	0.93

Supplementary Table 4. The skin & blood clock shares 45 CpGs (out of 71 CpGs) with the blood-based clock from Hannum (2013) and 60 CpGs (out of 353 CpGs) with the pan tissue clock from Horvath (2013). Specifically, it shares the following 45 CpGs with Hannum:

cg20822990 cg25410668 cg16054275 cg10501210 cg09809672 cg02085953 cg06639320 cg22454769 cg23606718 cg04474832 cg03607117 cg07553761 cg25478614 cg02650266 cg08234504 cg23500537 cg20052760 cg16867657 cg22736354 cg06493994 cg06685111 cg20426994 cg08097417 cg03473532 cg08540945 cg16419235 cg07583137 cg22796704 cg23091758 cg04940570 cg06419846 cg00748589 cg19722847 cg01528542 cg03032497 cg04875128 cg21296230 cg07082267 cg06874016 cg02867102 cg19283806 cg14556683 cg07547549 cg05442902 cg08415592

and it shares the following 60 CpGs with Horvath 2013:

cg05675373 cg01459453 cg02275294 cg21870884 cg09809672 cg22809047 cg10376763 cg10523019 cg12941369 cg26614073 cg15988232 cg03019000 cg04474832 cg03891319 cg14423778 cg05960024 cg25148589 cg10345936 cg23517605 cg01570885 cg22736354 cg06493994 cg22679120 cg13931228 cg20692569 cg04084157 cg14175438 cg04528819 cg19724470 cg07158339 cg01560871 cg04268405 cg04126866 cg13547237 cg01820374 cg00431549 cg13302154 cg19722847 cg18573383 cg10281002 cg09646392 cg06738602 cg24058132 cg02071305 cg21801378 cg02331561 cg22947000 cg06144905 cg25809905 cg17589341 cg26005082 cg26842024 cg24834740 cg27544190 cg01262913 cg17274064 cg12373771 cg05442902 cg19853760 cg23124451.
